# A Critical Perspective on 3D Liver Models for Drug Metabolism and Toxicology Studies

**DOI:** 10.3389/fcell.2021.626805

**Published:** 2021-02-22

**Authors:** Ana S. Serras, Joana S. Rodrigues, Madalena Cipriano, Armanda V. Rodrigues, Nuno G. Oliveira, Joana P. Miranda

**Affiliations:** ^1^Research Institute for Medicines (iMed.ULisboa), Faculty of Pharmacy, Universidade de Lisboa, Lisbon, Portugal; ^2^Fraunhofer Institute for Interfacial Engineering and Biotechnology IGB, Stuttgart, Germany

**Keywords:** *in vitro* liver model, fit-for-purpose models, hepatotoxicity, paracetamol, diclofenac, troglitazone, three-dimensional culture

## Abstract

The poor predictability of human liver toxicity is still causing high attrition rates of drug candidates in the pharmaceutical industry at the non-clinical, clinical, and post-marketing authorization stages. This is in part caused by animal models that fail to predict various human adverse drug reactions (ADRs), resulting in undetected hepatotoxicity at the non-clinical phase of drug development. In an effort to increase the prediction of human hepatotoxicity, different approaches to enhance the physiological relevance of hepatic *in vitro* systems are being pursued. Three-dimensional (3D) or microfluidic technologies allow to better recapitulate hepatocyte organization and cell-matrix contacts, to include additional cell types, to incorporate fluid flow and to create gradients of oxygen and nutrients, which have led to improved differentiated cell phenotype and functionality. This comprehensive review addresses the drug-induced hepatotoxicity mechanisms and the currently available 3D liver *in vitro* models, their characteristics, as well as their advantages and limitations for human hepatotoxicity assessment. In addition, since toxic responses are greatly dependent on the culture model, a comparative analysis of the toxicity studies performed using two-dimensional (2D) and 3D *in vitro* strategies with recognized hepatotoxic compounds, such as paracetamol, diclofenac, and troglitazone is performed, further highlighting the need for harmonization of the respective characterization methods. Finally, taking a step forward, we propose a roadmap for the assessment of drugs hepatotoxicity based on fully characterized fit-for-purpose *in vitro* models, taking advantage of the best of each model, which will ultimately contribute to more informed decision-making in the drug development and risk assessment fields.

## Introduction

The process of development of new drugs is a costly investment with the pharmaceutical industry facing considerable challenges regarding the balance between the political pressure to increase drugs safety while reducing the cost of medicines. According to a recent study by Wouters et al. (2020), the median investment of bringing a new drug into the market, also accounting for failed trials, was estimated at $985.3 million over the period of 2009–2018. It is a process that usually takes 10–15 years, with a success rate from phase I to launch of less than 10% (Dowden and Munro, 2019). This is mostly due to lack of drug efficacy or safety issues that occur essentially in the clinical phases IIb and III of drug development (Kola and Landis, 2004; Paul et al., 2010). Even after reaching the market (phase IV), there is still a relevant number of drug withdrawals for toxicological reasons. Approximately 18–30% of such withdrawals are caused by hepatotoxic effects, showing that the liver is the most frequent organ for adverse drug reactions (ADRs) (Onakpoya et al., 2016; Siramshetty et al., 2016; Zhang X. et al., 2020). Importantly, about 40–50% of the drug candidates associated with hepatotoxicity in humans did not present the same toxicological concern in animal models (van Tonder et al., 2013). Indeed, besides raising ethical issues, animal models often fail to correlate with human toxicity, since several toxic features disclosed in human trials were not predicted by animal studies (Olson et al., 2000; Shanks et al., 2009). One of the reasons for this discrepancy is the differential expression and activity of drug metabolizing enzymes between animals and humans that might confound the extrapolation of data derived from non-clinical species (Martignoni et al., 2006; Ruoß et al., 2020). Moreover, drug-induced liver injury (DILI) is a rare, but potentially fatal event, resultant from the poor translation between clinical trials and clinical practice and highlights the importance of targeting population variability at non-clinical stages (Jones et al., 2018). Within DILI, the idiosyncratic category is particularly difficult to identify by the pharmaceutical industry as it is almost undetectable in animal models (Kuna et al., 2018; Walker et al., 2020). Altogether, this has led to the proposal that the better the quality of non-clinical safety profiles, the higher the success rates for moving phase II upward (Cook et al., 2014; Walker et al., 2020). Consequently, *in vitro* liver models are growing strong while new drugs advance into clinical trials.

The search for more accurate non-clinical models along with the concern about animal welfare, reducing time and cost associated to drug development and the ever-increasing number of chemicals that need testing, made the establishment of relevant *in vitro* culture systems a priority in the toxicology assessment of drugs by the pharmaceutical industry, as these allow a higher-throughput capacity. Novel cell culture and tissue engineering technologies along with integrated endpoints have been adopted for improving liver cell metabolic performance *in vitro* and are expected to generate more robust data on the potential risks of pharmaceuticals (Davila et al., 2004; Andersen and Krewski, 2009, 2010; Krewski et al., 2009; Giri et al., 2010; Shukla et al., 2010; Balls, 2011; Mandenius et al., 2011). Existing strategies include three-dimensional (3D) structures, flow-based cultures, co-cultures and stem-cell differentiation.

In this review, we discuss the dissimilarities of the 3D *in vitro* hepatic systems currently used in research and drug development and their actual contribution for unraveling the mechanisms of drug-induced hepatotoxicity. Special emphasis is given to the features of 3D culture systems, cell organization and architecture, the effects of stirring and perfusion and how these characteristics modulate the phenotype and functionality of liver cells. In addition, we take a step forward by presenting a comparative analysis of the IC_50_ values for cytotoxicity and mechanistic endpoints, obtained either with two-dimensional (2D) and 3D *in vitro* systems for the classical hepatotoxic drugs paracetamol (acetaminophen), diclofenac and troglitazone. In this context, it seems clear the need for harmonized and fully characterized models. Moreover, it is also important to highlight that the hepatotoxicity assessment and the choice of the *in vitro* liver models depend on the questions that need to be addressed. These strategies stand out as crucial when evaluating the model’s relevance value for mechanism-based hepatotoxicity assessment.

## Drug-Induced Hepatotoxicity: Overview, Liver Metabolism and Mechanisms of Toxicity

The liver is responsible for most of the metabolism of orally administered drugs since its anatomical proximity to the gastrointestinal tract and histological structure, including the sinusoidal space and the blood supply from the portal vein, allows the efficient transport of drugs and other xenobiotics (Vernetti et al., 2017). It is a complex organ composed by ∼60% of hepatocytes, parenchymal cells responsible for multiple functions, including metabolism. Non-parenchymal cells (NPCs) include cholangiocytes lining the bile ducts; sinusoidal endothelial cells, which constitute a permeable barrier between the blood and the space of Disse; Kupffer cells, the liver-resident macrophages; and stellate cells, which synthesize fat and produce vitamin A and collagen (Kuntz and Kuntz, 2008).

Drug-induced hepatotoxicity is defined as the hepatic damage caused by the exposure to prescription-only or over-the-counter medicines, herbs or other xenobiotics. DILI represents a major challenge for clinicians, the pharmaceutical industry, and regulatory agencies worldwide. As above mentioned, it corresponds to the leading cause of attrition of compounds in drug development, being also frequently associated to drug withdrawals from market or to use restrictions (Stevens and Baker, 2009; Devarbhavi, 2012; Jones et al., 2018).

Classically, DILI can be classified as intrinsic (e.g., caused by paracetamol) or idiosyncratic (e.g., caused by troglitazone) hepatotoxicity. Intrinsic hepatotoxicity is direct, dose-dependent and predictable, whereas idiosyncratic hepatotoxicity occurs without obvious dose-dependency, in an unpredictable fashion and with a short latency time, particularly after re-exposure (Russmann et al., 2009; Roth and Ganey, 2010). Idiosyncratic DILI can be an allergic immune-mediated hypersensitivity or the result of a non-allergic metabolic injury (Larson, 2010). DILI may also be categorized according to the duration (i.e., acute or chronic) and location/typology of the injury. The latter can be classified as hepatitis (mostly due to hepatocyte necrosis), cholestatic (i.e., bile duct damage or cholangiolitis) or mixed injury (Stefan and Hamilton, 2010). Despite the variety of its clinical presentations, DILI still does not display specific biomarkers, leading to abnormal liver tests and often the dysfunction is only identified by exclusion of other etiologies, which can lead to life-threatening clinical situations (Devarbhavi, 2012; Fu et al., 2020). Indeed, the identification of new molecular biomarkers has been investigated in order to improve diagnosis and treatment of DILI. However, its applicability is still limited (Fu et al., 2020). Thus, DILI is largely unrecognized and underreported, such that the true incidence is unknown.

There are several examples of clinically relevant drugs that have received prescription restrictions or the inclusion of a black box warning for potential hepatotoxicity. Among hepatotoxic drugs, paracetamol is the most frequently studied. Nevertheless, the most commonly hepatotoxicity-associated pharmacological groups of orally administrated drugs are antibiotics (e.g., amoxicillin-clavulanate and rifampicin), antiretrovirals (e.g., nevirapine), non-steroidal anti-inflammatory drugs (NSAIDs, e.g., diclofenac and ibuprofen), antidepressants (e.g., paroxetine), and anticonvulsants (e.g., phenytoin, carbamazepine, and valproic acid) (EMEA, 2000; Paniagua and Amariles, 2018). Among intravenous administration, antibiotics, and antineoplastic drugs are the pharmacological groups mostly associated with hepatic toxicity (Ghabril et al., 2013). It should be mentioned that during the past decades, particularly in the last 20 years, several medicines such as troglitazone, bromfenac, trovafloxacin, ebrotidine, nimesulide, nefazodone, ximelagatran, lumiracoxib, pemoline, tolcapone, and sitaxentan have also been removed from the market in some countries in Europe and in the United States due to severe DILI (Fung et al., 2001; Qureshi et al., 2011; Babai et al., 2018).

### Liver Metabolism

Drug metabolism is a major determinant of hepatotoxicity, as both detoxification and bioactivation processes can occur, and are most frequently responsible for inter-individual differences in drug-induced toxicity.

**FIGURE 1:**
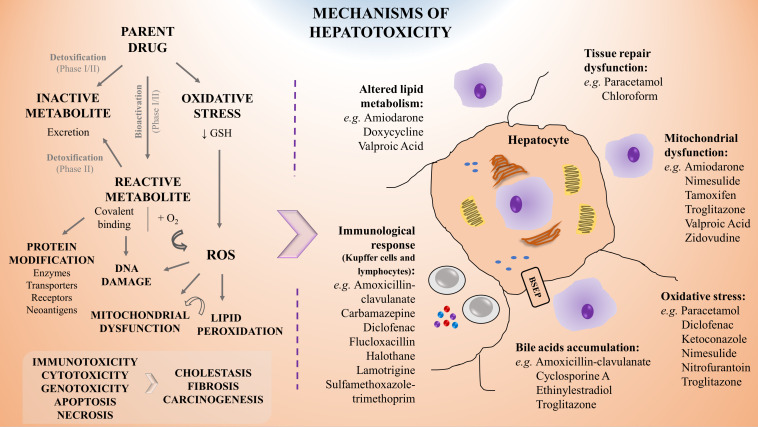
Schematic representation of the mechanisms of hepatotoxicity including examples of associated drugs. Drug biotransformation (phase I and II metabolism) is based on the chemical modification of a parent drug into a metabolite which may become inactive (detoxification), leading to its rapid and innocuous excretion, or reactive (bioactivation), leading to potential toxicity. Specifically, hepatotoxicity may result from direct damage, from failure of repairing mechanisms or from immune-mediated responses, leading to alterations in lipids metabolism, mitochondrial dysfunction, oxidative stress and accumulation of bile, amongst others. Moreover, the saturation of cells stress defense mechanisms may lead to carcinogenic events and promote tissue necrosis or fibrosis, resulting in liver’s functions impairment. For a given hepatotoxic compound different mechanisms of toxicity can be involved. GSH, reduced glutathione; ROS, reactive oxygen species.

Liver metabolism encompasses phase I biotransformation reactions, also known as functionalization reactions, leading to the hydrolysis, oxidation, and reduction of a given drug or xenobiotic. Key enzymes in this phase belong to the CYP450 family, but can also be epoxide hydrolase and monoamine oxidase, among others. The metabolites generated can be detoxified or bioactivated by further phase I biotransformation or by conjugation through phase II metabolism (e.g., glucuronidation, sulfation, and acetylation). The role of liver transporters (e.g., organic anion-transporting polypeptides, OATP, multidrug resistance-associated proteins, and MRP) is of great importance for the excretion, being this step also known as phase III (Gomez-Lechon et al., 2010; Yuan and Kaplowitz, 2013). A significant feature of liver drug metabolism is that it may transform the parental compounds into chemically reactive intermediates or electrophilic metabolites (i.e., bioactivation) that attack tissue constituents, potentially leading to mutations, cancer or tissue necrosis (Pessayre, 1993). Drug-induced hepatotoxicity can thus be consequence of the toxicity of the parental drug *per se* or the result of one or more of its metabolites that arise from liver metabolism (Figure 1). Therefore, the toxicity of a given xenobiotic greatly depends on the equilibrium between detoxification and bioactivation. Hence, in a new drug development scheme, the biotransformation processes should be widely studied in order to predict the physiological effect of the new compound.

There are several prodrugs that take advantage of liver metabolism, e.g., cyclophosphamide (Preissner et al., 2015) and L-Dopa (Di Stefano et al., 2011), as the initial molecule is only active after biotransformation near the target site, decreasing its potential toxicity and also increasing its bioavailability. On the other hand, paracetamol is an interesting example in which hepatotoxicity is dose-dependent and occurs since its metabolic pathway switches at a high dose exposure from the detoxifying phase II metabolism to phase I metabolism, generating the hepatotoxic metabolite *N*-acetyl-*p*-benzoquinone imine (NAPQI). This metabolite can covalently react with proteins, leading to necrosis, apoptosis and, ultimately, to liver failure (Hinson et al., 2010). Additionally, phase II metabolism may also lead to hepatotoxic derivatives, such as for example carboxylic acids, e.g., bromfenac (and other NSAIDs) or valproic acid (Sidenius et al., 2004; Skonberg et al., 2008). These can be bioactivated to acyl-coenzyme A thioesters, which are intermediates in phase II conjugation reaction, and may lead to reactivity toward reduced glutathione (GSH) and covalent binding to endogenous proteins (Sidenius et al., 2004; Skonberg et al., 2008). Hence, factors including the inhibition or induction of any of the biotransformation enzymes, drug-drug interactions, or genetic polymorphisms, may lead to increased activity and toxicity or, on the other hand, to an absence of effect.

Several widely prescribed drugs are themselves potent CYP450 enzyme inducers, e.g., phenobarbital, carbamazepine and rifampicin, or inhibitors, e.g., fluoxetine, ritonavir, fluconazole, and ciprofloxacin (Baxter et al., 2010; Wooten, 2015; Wolverton and Wu, 2020). Subsequently, in the context of multiple drug prescription, the biotransformation of drugs that are substrates of CYP450 enzymes or other phase II enzymes and hepatic transporters can be severely altered when administered simultaneously. Some antiretroviral drugs, such as efavirenz (Grilo et al., 2017) or nevirapine (Pinheiro et al., 2017), may be simultaneously the substrate and the inducer of an enzyme, such as CYP3A4 and CYP2B6, and can regulate its own biotransformation (auto-inducer) (Kappelhoff et al., 2005). Indeed, enzyme induction is included within the pharmacokinetic (PK) tolerance concept, as it can lead to overdose reactions (higher parent drug/metabolite activation) or to sub-therapeutic exposures (lower parent drug/metabolite inactivation) to drugs when normal doses are administered (Dumas and Pollack, 2008; Omiecinski et al., 2011; Jaeschke, 2013). However, the effect that xenobiotics can exert on the induction or inhibition of biotransformation enzymes is especially difficult to predict with the currently existing *in vitro* and *in vivo* drug testing models, mainly due to interspecies and inter-individual differences, or decreased cells functionality (Reder-Hilz et al., 2004; Zanger et al., 2007; Godoy et al., 2013).

Genetic polymorphisms are particularly relevant risk factors regarding drug-metabolizing enzymes and may represent susceptibility biomarkers, important for predicting potential hepatotoxicity risks. Genetic polymorphisms are common gene variations that might encode for impaired/altered metabolic enzymes and generate different population subgroups in terms of metabolism assessment (Meyer and Zanger, 1997; Andrade et al., 2009; Ahmad and Odin, 2017). As this event is not rare, these subgroups need to be accounted in a drug development scheme and, thus, properly mimicked at the non-clinical stage. Interindividual variability concerning phase I, II, and III enzyme expression can also justify some cases of hepatotoxicity. Genetic polymorphisms are reported to affect the biotransformation of drugs dependent on CYP2C9, CYP2C19, CYP2B6, CYP2D6, CYP3A4, and CYP3A5 subfamilies, phase II enzymes uridine 5′-diphosphate glucuronosyltransferase (UGT) 1A1, UGT2B7 and *N*-acetyltransferase (NAT) 2, and hepatic transporters multidrug resistance protein (MDR) 1, breast cancer resistance protein (BCRP), MRPs, and OATP1B1, amongst others (Wienkers and Heath, 2005; Zanger et al., 2007; Brockmöller and Tzvetkov, 2008; Shah et al., 2015; Krasniqi et al., 2016; Saiz-Rodríguez et al., 2020). Some classical examples include CYP2D6, due to its clinical impact in the bioactivation of drugs such as codeine, tramadol, or tamoxifen within low or extensive metabolizers (Cavallari et al., 2019). Another classical example are NAT2 polymorphisms, reflected in slow, intermediate, and rapid acetylators of drugs, particularly isoniazid (anti-tuberculosis drug), in which the former presents potentially more ADRs than the latter (Brockmöller and Tzvetkov, 2008). Moreover, an inherited mutation in the adenosine triphosphate (ATP)-binding cassette subfamily B (ABCB) 11 gene, which encodes for bile salt export pump (BSEP), may lead to the diminishing of the bile acids transport and clearance, potentially leading to cholestasis (Kenna and Uetrecht, 2018).

### Mechanisms of Hepatotoxicity

The liver is a prime target for drug-induced damage due to its central role for concentrating and metabolizing the majority of drugs. Therefore, earlier and better understanding of drug modes of action and toxicity are essential (Kola and Landis, 2004; Paul et al., 2010; Padda et al., 2011). As above mentioned, following exposure, the toxic effect of a given drug may be attributed directly to the interaction of the parent drug or the product of its biotransformation, with an endogenous target through covalent or non-covalent binding, hydrogen abstraction, electron transfer, or enzymatic reactions, resulting in dysfunction or destruction of the target molecules (Chan and Benet, 2017). Moreover, besides arising from direct damage by the molecule, hepatotoxicity may also be resultant from a failure of repair mechanisms or due to immune-mediated responses. The mechanisms of hepatotoxicity more frequently described are depicted in Figure 1 and involve:

i) Mitochondrial dysfunction, an effect that may occur upon the exposure to different drugs, particularly amiodarone (Bethesda, 2012), nimesulide (Singh et al., 2010), troglitazone (Smith, 2003), or valproic acid (Xu et al., 2019);
ii) Oxidative stress, as observed for instance upon paracetamol or nitrofurantoin administration (Bethesda, 2012; Bruderer et al., 2015; Ramachandran and Jaeschke, 2018);
iii) Covalent binding with proteins that may impair their transporter function leading to accumulation of toxic elimination products and intrahepatic cholestasis (Boelsterli, 2003; Padda et al., 2011), as reported for ethinylestradiol and cyclosporine (Bethesda, 2012). It may also alter their conformation or structure as observed on the inhibition of hepatic synthesis of coagulation factors by exposure to coumarins (Grattagliano et al., 2009; Gregus, 2013);
iv) DNA damage, as suggested in the context of nevirapine toxicity (Kranendonk et al., 2014; Pinheiro et al., 2017; Marinho et al., 2019);
v) Depletion of enzymes or co-factors as observed upon paracetamol overdose (Mazaleuskaya et al., 2015; Ramachandran and Jaeschke, 2018);
vi) Dysfunction of cell repairing mechanisms that can result in: tissue necrosis, as for example by sulfasalazine, ketoconazole, or valproic acid (Kleiner, 2017); in fibrosis, by e.g., chronic exposure to methotrexate, high doses of retinol (vitamin A), and iron intoxication (Zhang et al., 2016); or in carcinogenesis, as a consequence of aflatoxin B1 exposure (Gregus, 2013; Jaeschke, 2013; Cai et al., 2020);
vii) Immunological-mediated tissue damage, that has been linked to NSAIDs such as diclofenac (Aithal et al., 2004), antibiotics such as amoxicillin-clavulanate (Bethesda, 2012) or flucloxacillin (Woolbright and Jaeschke, 2018) and anticonvulsants such as carbamazepine or lamotrigine (Bethesda, 2012).

These molecular mechanisms may intersect with each other leading to a cascade of key events. Indeed, an initial drug-related reactive oxygen species (ROS) formation may lead to lipid peroxidation on fatty acids chains in the cell membrane. In parallel, β-oxidation of lipids and oxidative stress may cause mitochondrial membrane permeabilization and dysfunction, ultimately leading to hepatocyte apoptosis. The rupture of the mitochondrial membrane can result in ATP depletion that accompanied by an increase in intracellular calcium concentration may generate liver necrosis. Conversely, inhibition of peroxisomal fatty acid β-oxidation may result in abnormal triglycerides accumulation in the hepatocyte and result in liver steatosis (Gregus, 2013). Adverse outcome pathways (AOPs) are promising tools in that regard, as they describe existing knowledge concerning the linkage between a direct molecular initiating event (MIE) and an adverse outcome through a number of key events (KEs) at a biological level of organization relevant to risk assessment (Gijbels and Vinken, 2017).

At the cellular level, the paracrine communication between hepatocytes and NPCs is also crucial for the response to a toxic insult. It has been reported that NPCs, after a primary injury of the hepatocyte, exhibit a secondary response that may aggravate or ameliorate the initial lesion, e.g., metabolic alterations and activation of immune cells, such as Kupffer cells and lymphocytes (Figure 1; Godoy et al., 2013; Kostadinova et al., 2013; Messner et al., 2013; Leite et al., 2016; Proctor et al., 2017; Bell et al., 2020; Li et al., 2020).

## Liver *in vitro* Models for Toxicological Studies

Both liver metabolism and the mechanisms of initial liver injury are important to comprehend the potential toxicity of a drug. Therefore, the development of efficient and fit-for-purpose *in vitro* models should mimic the complexity of the *in vivo* hepatic milieu. As such, when building a relevant liver *in vitro* model, the hepatic cell sources and tissue architecture, flow dynamics and the formation of molecular gradients need to be carefully considered.

No universally accepted hepatocyte source that provides robust, predictive and significant toxicological and pharmacological results is currently available. Cell source selection depends on cell availability and study requirements while understanding the limitations associated to each cell origin, namely metabolic competence, stability, and population representativeness (Soldatow et al., 2013). Regarding culture architecture, efforts have been focused in better mimic the *in vivo* microenvironment, giving special attention to culture three-dimensionality either by taking advantage of cell self-assembling capacity or by using natural polymers. More complex systems, such as bioreactors, micropatterning techniques, or microfluidic devices can also be employed (Miranda et al., 2010; Bell et al., 2016; Knospel et al., 2016; Adiels et al., 2017). Those platforms should also allow acute toxicity studies and long-term assessment so that the exposure to a xenobiotic generates relevant responses (Jiang et al., 2019). Overall, the value of an *in vitro* model depends on how well it reproduces the key physiological characteristics of an *in vivo* system. However, the criteria for defining liver function maintenance *in vitro* are not consensual, ranging from focusing on the preservation of hepatocyte phase I and II enzyme functions to the inclusion of a broader spectrum of tissue characteristics involved in human liver toxicity, such as the incorporation of NPCs for mimicking cells’ crosstalk (Bale et al., 2014; Zeilinger et al., 2016; Langhans, 2018; Bell et al., 2020).

Some common evaluated features to compare hepatic cell-based *in vitro* culture systems’ value for toxicological applications include cell morphology, viability, and functional stability; metabolic capacity; preservation of hepatic-specific gene expression under long-term cultures; and response to a panel of well-accepted reference drugs (e.g., paracetamol and valproic acid) capable of replicating human *in vivo* intrinsic DILI (Miranda et al., 2009, 2010; Leite et al., 2011; Mueller et al., 2011; Tostoes et al., 2011; Cipriano et al., 2017b; Pinheiro et al., 2017; Vinken and Hengstler, 2018; Bell et al., 2020). Moreover, the generated data should be able to be correlated to clinical observations, reproducible, comparable among laboratories, and analyzed properly to support decision-making with a clear definition of the models’ applicability and limitations (Dash et al., 2009; Vinken and Hengstler, 2018; Albrecht et al., 2019).

### Liver Cell-Based Versus Stem Cell-Based Models

Over the past decades, large efforts have been made to establish predictive *in vitro* liver test models. However, despite the number of reports available, a comprehensive and systematic comparison between cell culture systems adequate to objectively rank or select them for pharmacological and toxicological applications is still scarce.

**FIGURE 2:**
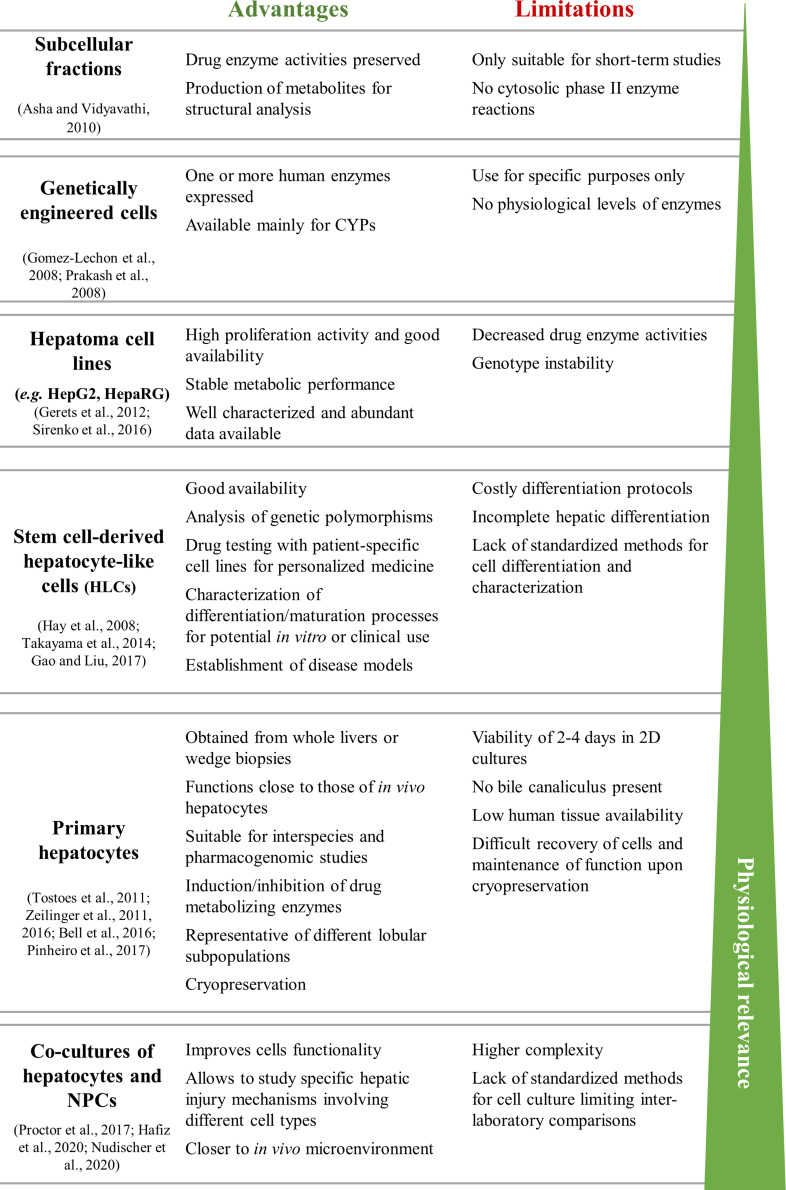
Summary of the advantages and limitations of commonly used cell sources for *in vitro* liver models. HLCs, hepatocyte-like cells; hpHep, human primary hepatocytes; NPCs, non-parenchymal cells.

Several *in vitro* human-based models for the prediction of hepatotoxicity have been developed using a range of cell sources and endpoints. These include the use of liver slices, genetically engineered cells, human hepatoma cell lines (e.g., HepG2, THLE, and HepaRG cells), primary hepatocytes or stem cell (SC)-derived models (Gomez-Lechon et al., 2008; Asha and Vidyavathi, 2010; Sirenko et al., 2016; Gao and Liu, 2017; Pinheiro et al., 2017; Nudischer et al., 2020). Figure 2 summarizes the advantages and limitations of each cell source for *in vitro* testing, as well as their *in vivo* physiological relevance.

Liver slices and isolated perfused livers, containing both parenchymal and NPCs, retain liver’s structure and thus maintain zone-specific enzymatic activity. However, within hours, the cell functionality decreases and necrosis takes place (Lerche-Langrand and Toutain, 2000; Boess et al., 2003; Haschek et al., 2009). It is associated with limited throughput and requires continuous animal experimentation and personnel expertise (Vernetti et al., 2017).

Alternatively, cell-based models are less complex and associated to higher throughput screening for the identification of hepatotoxic compounds. Primary hepatocytes, either obtained from human liver autopsies or biopsies or from animal livers, have been used for cytotoxicity, biotransformation, and PK studies (Vernetti et al., 2017). Human primary hepatocytes (hpHep), in particular, are considered the gold standard in human-relevant liver *in vitro* models for cytotoxicity and drug metabolism testing, retaining most of the native tissue’s functionality, namely phase I and phase II enzymes (Godoy et al., 2013; Zeilinger et al., 2016). However, both the limited availability of primary human cells and its suitability only for short-term studies under monolayer cultures are major disadvantages. Indeed, in 2D conditions, it is observed a progressive loss of the hepatic phenotype in a process called de-differentiation, which is a consequence of the disruption of cell–cell and cell-matrix connections (Zeilinger et al., 2016). Additionally, hpHep display inter-donor variability and thus the use of different cell batches to validate results is advised, covering several metabolic genetic polymorphism and phenotypes (Godoy et al., 2013; Zeilinger et al., 2016). On the other hand, rat primary hepatocytes (rpHep), despite being more easily available, present relevant interspecies differences (Sandker et al., 1994; Li et al., 2008; Ménochet et al., 2012; Shen et al., 2012).

Human hepatoma cell lines, such as HepG2 and HepaRG, have no limitations in terms of cell numbers and are easy to culture, but display poor phenotype and functional match to *in vivo* hepatocytes (Gerets et al., 2012). The use of these cell lines do not consider populational differences and may reflect characteristics that primary cells do not have, e.g., beingmore sensitive to compounds with anti-proliferative properties (Sirenko et al., 2016). HepG2 present low levels of CYPs and normal levels of phase II enzymes except for UGTs (Westerink and Schoonen, 2007a, b), which make them appropriate for testing the toxicity of the parent compound but less suited for metabolite toxicity testing. Instead, HepaRG cell line composed of a mixture of both hepatocyte-like and biliary-like cells, have been reported to maintain hepatic functions and expression of liver-specific genes comparable to hpHep without the inter-donor variability and functional instability issues (Guillouzo et al., 2007). Nevertheless, it should be noted that a cell characterization study at the mRNA/gene expression and CYP activity levels, by Gerets et al. (2012), revealed that although it is a suitable model for induction studies, these cells were not as indicative as hpHep for the prediction of human hepatotoxic drugs, being comparable to HepG2 cells. On the other hand, Lübberstedt et al. (2011) showed that HepaRG presented similar or even higher CYP2C9, CYP2D6, and CYP3A4 enzyme activity than that of hpHep, whereas Aninat et al. (2006) confirmed the presence of relevant UGT1A1 and GST activity levels. Still, high metabolic capacity in cell lines does not necessarily correlate with high sensitivity for the hepatotoxicity detection (Gerets et al., 2012). Thus, unfortunately, even the most promising and differentiated hepatoma cells do not constitute an ideal surrogate system for human hepatocytes for hepatotoxicity studies, as they do not reproduce the drug-metabolizing enzyme pattern of human hepatocytes. An alternative approach to overcome the limitations of hepatic cell lines is to genetically modify cells with vectors encoding for human CYP enzymes and other genes involved in xenobiotic metabolism (Coecke et al., 2001; Kanamori et al., 2003; Gomez-Lechon et al., 2008; Prakash et al., 2008; Godoy et al., 2013). However, the number of enzymes that can be satisfactorily transfected into cells is low and the metabolic profiles differ from those of primary hepatocytes (Frederick et al., 2011; Godoy et al., 2013).

To overcome the limitations of the above mentioned cell sources, SC-derived human hepatocyte-like cells (HLCs) have been suggested as a reliable alternative (Szkolnicka et al., 2014; Takayama et al., 2014; Freyer et al., 2016; Cipriano et al., 2017a, b, 2020; Figure 2). SCs represent normal primary cells with a mostly stable genotype than hepatoma cell lines. Moreover, compared to hpHep, present unlimited supply, can be maintained for long-term and may also represent a broad patient population (Godoy et al., 2013; Horvath et al., 2016). As such, stem or progenitor cells are an exciting prospect for drug metabolism studies and cell transplantation, providing that high levels of hepatocyte-like functions can be induced and tumorigenicity concerns are overcome. Many protocols have been developed for differentiating SCs into HLCs with different approaches, such as mimicking liver development through the sequential addition of growth factors and cytokines (Cai et al., 2007; Hay et al., 2008b; Brolén et al., 2010), modulation of signaling pathways (Hay et al., 2008a) or by using epigenetic modifiers (Sharma et al., 2006; Dong et al., 2009; Norrman et al., 2013). Currently, most work has been developed using induced pluripotent SCs (iPSCs) isolated from adult tissues in an non-invasive way, with promising outcomes (Sauer et al., 2014; Sirenko et al., 2016; Yamashita et al., 2018; Pareja et al., 2020). An example is the work from Gao and Liu (2017), that revealed that iPSC-derived HLCs resembled hpHep more closely than most hepatoma cell lines in global gene expression profiles, specifically in the expression of genes involved in hepatotoxicity, drug-metabolizing enzymes, transporters, and nuclear receptors. Interestingly, Freyer et al. (2016) detected CYP1A2, CYP2B6, and CYP3A4 activities in iPSC-derived HLCs, but also at a lower level than in hpHep. Likewise, Takayama et al. (2014) showed that iPSC-derived HLCs retained donor-specific drug metabolism capacity and drug responsiveness, reflecting interindividual differences, but lower CYP1A2, CYP2C9, CYP2D6, and CYP3A4 activities when compared to the correspondent hpHep donors. Besides hepatocytes, efforts have also been made to generate NPCs from iPSCs, including cholangiocytes (Ogawa et al., 2015; Sampaziotis et al., 2015), Kupffer cells (Tasnim et al., 2019), LSECs (Koui et al., 2017), and hepatic stellate cells (Koui et al., 2017; Coll et al., 2018). Nevertheless, iPSC technology has some limitations related to the genomic instability and to residual iPSC-specific methylation patterns that links these cells to their tissue of origin, which ultimately may affect their final differentiation (Robinton and Daley, 2012). Still, iPSC-derived HLCs show powerful value not only for toxicology applications but also for disease modeling and personalized drug therapy.

Alternatively, adult liver SCs (LSCs) are a particularly interesting SC source. LSCs can be obtained from liver biopsies, propagated *in vitro* and differentiated into mature hepatocytes (Huch et al., 2015; Wang et al., 2015; Luo et al., 2018). LSCs are located in the epithelium of the canals of Hering and contribute to liver regeneration in response to an injury (Overi et al., 2018). LSCs are bipotent, being able to differentiate into hepatocytes or cholangiocytes. As such, these cells express SC (e.g., SRY-box transcription factor 9, Sox9), cholangiocyte (CK-19), and hepatocyte (CK-18) markers (Overi et al., 2018). The identification of populations of proliferating and self-renewing cells that can replace injured hepatocytes can be performed with lineage tracing approaches using *Wnt*-responsive genes such as *Axin2* or *Lgr5* (Huch et al., 2013; Wang et al., 2015).

Mesenchymal SCs (MSCs) including liver, bone-marrow, adipose, or umbilical cord tissue-derived MSCs have also been used for deriving human HLCs (Snykers et al., 2006, 2007; Banas et al., 2007; Kazemnejad et al., 2008; Okura et al., 2010; Yin et al., 2015; Fu et al., 2016; Yang et al., 2020). From those, human neonatal MSCs stand as a promising choice due to the non-invasive access and to its more primitive origin (Hass et al., 2011; Lee et al., 2012; Cipriano et al., 2017a; Yu Y. B. et al., 2018. The first report using human neonatal umbilical cord tissue-derived MSCs (hnMSCs) was from Campard et al. (2008). Therein, hnMSCs were differentiated into HLCs with impressive results, i.e., presenting hepatic-specific markers, urea production, glycogen accumulation, and CYP3A4 activity. Afterward, other researchers also differentiated hnMSCs into HLCs exhibiting hepatic markers, urea and albumin (ALB) production. However, their biotransformation activity was not assessed (Zhang et al., 2009; Zhao et al., 2009; Zhou et al., 2014). More recently, Cipriano et al. (2017a) generated hnMSC-derived HLCs with more partial hepatic phenotype, sharing expression of gene groups with hpHep that was not observed between HepG2 and hnMSCs, as shown by genome-wide analysis (Cipriano et al., 2017a). Importantly, when resorting to the 3D culture technology, MSC-derived HLCs demonstrate an improvement in phase I biotransformation activity, urea and ALB production, as well as relevant diclofenac and nevirapine biotransformation capacity, which supports its potential usefulness for toxicological studies (Cipriano et al., 2017b, 2020). Nevertheless, despite the growing efforts made in this research field a complete mature hepatocyte phenotype of HLCs derived from MSCs has not yet been achieved. Perhaps liver MSCs may be the best choice, because they are originally committed to hepatic lineage, but an accurate comparison of hepatocytes derived from human liver MSCs and other sources must still be done (Kholodenko et al., 2019; Shi et al., 2020).

All these strategies are not deprived of challenges as they require specialized personnel and expensive culture medium supplementation, whereas a complete mature phenotype has not yet been achieved. The fetal HLC phenotype is still a challenge, revealing the need to further understand hepatic differentiation mechanisms and optimizing differentiation strategies (Raju et al., 2018; Raasch et al., 2019). Moreover, the use of diverse differentiation protocols across different laboratories hinders the robustness assessment of the use of HLCs for toxicology applications. To address this issue, some authors proposed a set of cellular markers and functional assays to control the quality of iPSC-derived cells, since these are the most common type of SCs used *in vitro* (Daston et al., 2015; Beken et al., 2016). Although the specific metrics to monitor cell characteristics may vary according to the differentiation protocol and cell line used, this guide provides an important reference for quality control of other types of SC-based models. For HLCs, the most important markers to be analyzed are CYP3A4, CYP2B6, CYP1A1/2, CYP2C9, CYP2C19, CYP2D6, alpha-fetoprotein (AFP), ALB, Sox17, C-X-C motif chemokine receptor 4 (CXCR4), hepatocyte growth factor (HGF), hepatocyte nuclear factor 4 alpha (HNF-4α), tyrosine aminotransferase (TAT), transthyretin (TTR) while functional assays include urea and ALB synthesis, glycogen uptake, fibrinogen secretion, ATP, and GSH levels, CYP3A activity in particular, phase II activities and drug transporter capacity (Beken et al., 2016). Nonetheless, due to overall unsatisfactory phenotype of the currently available cell sources, at least for some hepatic features, the improvement of the cell culture system has been explored as will be further described in the following sections.

### Three-Dimensional Liver Systems

The major shortcoming of the currently available *in vitro* liver preparations lays on insufficient hepatocyte-like functions and metabolic competence. In fact, none of the hpHep-, HepG2-, or HepaRG-based 2D models are suitable to indicate the risk of hepatotoxicity for novel chemical entities unless PK data are incorporated in the study, supporting the need to employ more sophisticated technologies to increase prediction sensitivity (Sison-Young et al., 2017). Accordingly, recent reports emphasize a shift, by the industry, from 2D *in vitro* approaches to more complex 3D assays where multicellular microphysiological devices are being evaluated within a vision to replicate the characteristics and response of human tissues *in vivo* (Vivares et al., 2015).

Traditionally, 2D cultures are employed as *in vitro* models due to their ease of use to quickly screen large numbers of compounds. However, this culture approach negatively impacts cell expression profiles (Engler et al., 2006) and causes primary hepatocytes to rapidly lose their differentiation markers (Treyer and Müsch, 2013), compromising long-term and repeated dose studies. On the other hand, 3D cell culture systems have been shown to improve the biotransformation capacities in primary hepatocytes (Tibbitt and Anseth, 2009; Miranda et al., 2010; Mandenius et al., 2011; Mueller et al., 2011; Zeilinger et al., 2011; Schyschka et al., 2013), hepatoma cell lines (Fey and Wrzesinski, 2012; Molina-Jimenez et al., 2012; Wrzesinski et al., 2014) and SC-derived HLCs (Gieseck et al., 2014; Freyer et al., 2016; Cipriano et al., 2017b, 2020) over time in culture.

**FIGURE 3:**
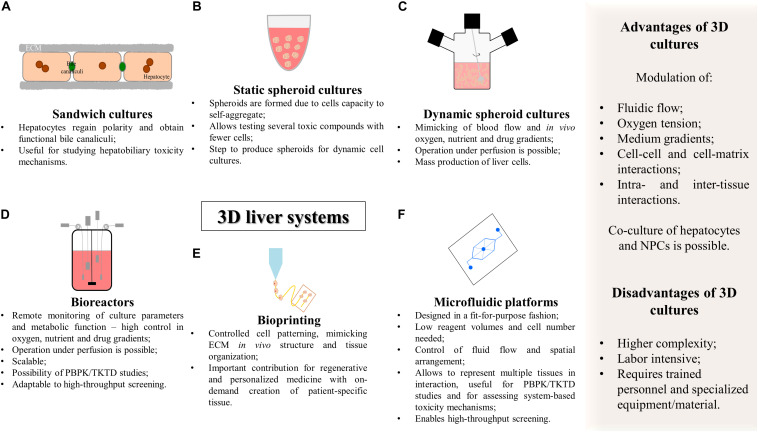
Summary of the characteristics of complex 3D *in vitro* cell culture systems for hepatotoxicity studies. **(A)** Sandwich cultures; **(B)** static spheroid cultures; **(C)** dynamic spheroid cultures; **(D)** bioreactors; **(E)** bioprinting; **(F)** microfluidic platforms. PBPK, physiologically based pharmacokinetic modeling; TD, toxicodynamics; TK, toxicokinetics.

In general, as summarized in Figure 3 and Table 1, **3D** cell culture systems are prone to high-throughput adaptation and scale up but vary in complexity and on remote monitoring of cell culture parameters. Three-dimensional systems can comprise extracellular matrix (ECM) sandwich cultures (Chatterjee et al., 2014; Deharde et al., 2016), spheroid and organoid cultures (Miranda et al., 2009; Leite et al., 2011, 2012; Tostoes et al., 2011; Wrzesinski et al., 2014; Huch et al., 2015; Bell et al., 2016; Peng et al., 2018; Ramli et al., 2020), cells adherent to a scaffold (Kazemnejad et al., 2008; Lin and Chang, 2008; Haycock, 2011), or more complex cellular systems such as hollow-fiber bioreactors (Darnell et al., 2011, 2012; Lübberstedt et al., 2011; Mueller et al., 2011; Zeilinger et al., 2011; Hoffmann et al., 2012; Cipriano et al., 2017b), bioartificial livers (Chan et al., 2004), multi-well perfused bioreactors (Domansky et al., 2010; Vivares et al., 2015; Aeby et al., 2018; Mannaerts et al., 2020), and more recently bioprinted systems (Lauschke et al., 2016; Goulart et al., 2019) and microfluidic platforms (MP) (Rennert et al., 2015; Ma C. et al., 2016; Bauer et al., 2017; Danoy et al., 2019).

**TABLE 1:** Advanced 3D liver cell models commercially available.

Commercial name	Cell model	Features	Studies employing the advanced liver model
LiverChip/PhysioMimix Liver-on-Chip	Human or rat primary hepatocytes cultured with NPCs (e.g., liver sinusoidal endothelial cells, LSECs; stellate and Kupffer cells)	Liver tissue-engineered perfused bioreactor; Viability for at least 1 month; Scalable; Allows to mimic liver zonation; Allows PBPK studies; Allows prediction of *in vivo* hepatic clearances; Expression of genes of phase I, phase II, and phase III similar to freshly thawed hepatocytes	Domansky et al., 2010; Vivares et al., 2015; Tsamandouras et al., 2017
HμREL Technology	Human, primate, dog or rat primary hepatocytes cultured with NPCs	Compartments designed to represent tissue-functional units (microfluidic devices); Viability for at least 10 days; Phase I and phase II enzymes activity similar to freshly thawed hepatocytes; Allows prediction of *in vivo* hepatic clearance, particularly with low clearance compounds; Enables multi-parametric and repeated-dose readouts	Chao et al., 2009; Bonn et al., 2016; Hultman et al., 2016; Novik et al., 2017
HepaPredict	Human primary hepatocytes cultured with Kupffer, stellate and biliary cells	Spheroids in well-plates; Viability for at least 35 days; Allows to study inter-individual variability; Expression of genes of metabolic enzymes, drug and bile transporters similar to isolated hepatocytes; CYP1A2, CYP2C8, CYP2C9, CYP2D6, and CYP3A4 activity; Polarized cellular organization with functional bile canaliculi; Enables studies of chronic toxicity, long-term metabolic analyses, enzyme induction assays, drug target validation and disease modeling	Bell et al., 2016, 2017; Hendriks et al., 2016; Vorrink et al., 2017
HepatoPac	Human, monkey, dog or rat primary hepatocytes supported by mouse embryonic 3T3 fibroblasts	Micropatterned plates; Viability for at least 4 weeks; Phase I, phase II, and phase III enzymes activity similar to primary hepatocytes; Allows prediction of *in vivo* hepatic clearance, particularly with low clearance compounds	Khetani and Bhatia, 2008; Wang et al., 2010; Chan et al., 2013; Kratochwil et al., 2018
3D InSightLiver Microtissues	Human, monkey, dog or rat primary hepatocytes cultured with Kupffer cells and LSECs	96-well format, 1 microtissue per well; Viability up to 28 days; Albumin secretion; Activity of cytochrome P450; Polarized cellular organization with functional bile canaliculi; High mitochondrial function; Allows hepatotoxicity assessment and disease modeling	Messner et al., 2013, 2018; Proctor et al., 2017
MIMETAS OrganoPlate^®^	Human primary hepatocytes, HepaRG, iPSC-derived, UpCytes, and HepG2 cultured with stellate cells, Kupffer cells, bile duct, and LSECs	Supports up to 96 tissues on a single well-plate; Viability for at least 2 weeks; Polarized cellular organization with functional bile canaliculi; Perfusion supports long-term culture and formation of adjacent LSECs tubular structure; Defined ECMs tailored to support liver cells; Allows PBPK studies; Allows disease modeling	Jang et al., 2015
Liver-Chip	Human, dog or rat primary hepatocytes cultured with NPCs	Microfluidic device; Viability up to 2 weeks; Albumin and urea secretion; CYP1A2, CYP2B6, and CYP3A4 activity; Polarized cellular organization with functional bile canaliculi; Allows energy metabolism studies; Allows the recapitulation of different hepatotoxicity mechanisms (e.g., steatosis, cholestasis, and fibrosis)	Jang et al., 2019

Three-dimensional cell cultures can also be achieved using either static or dynamic systems. Static systems are less complex and do not include medium flow, while dynamic systems might be stirred and/or perfused, depending on the cell culture system complexity (Miranda et al., 2009, 2010; Tostoes et al., 2011). Static culture systems are compliant with high-throughput and are usually adopted for the optimization of culture medium constitution, to test a diversity of toxic compounds using fewer cells or as a step to produce spheroids to be used in more complex 3D culture systems, e.g., bioreactors (Wrzesinski et al., 2014; Fey et al., 2020). In contrast, stirring conditions facilitate oxygen diffusion as well as medium homogenization, further resembling the physiological blood flow, and create a hydro-dynamic shear stress that must be balanced, by improving cell performance while avoiding cellular stress and death (Conway et al., 2009). Also, a continuously perfused system is particularly interesting in hepatocyte cell culture and in xenobiotic metabolism studies, avoiding fluctuations of basic cell culture parameters such as pH, oxygen, glucose, and lactate concentration and the accumulation of metabolites that influence the PK of a specific compound (Conway et al., 2009; Miranda et al., 2009; Shvartsman et al., 2009; Mueller et al., 2011; Tostoes et al., 2011; Vinci et al., 2011; Zeilinger et al., 2011; Lauschke et al., 2016; McCarty et al., 2016; Prodanov et al., 2016). In addition, stirred and/or perfused systems promote a liver-like mass transfer which might mimic liver zonation *in vitro* (Allen et al., 2005; McCarty et al., 2016; Tomlinson et al., 2019). This wide variety of studies employs distinct cell sources and distinct approaches for creating 3D culture systems, showing encouraging results for *in vitro* hepatotoxicity models.

#### Sandwich Cultures

The sandwich culture system consists of culturing hepatocytes between two layers of ECM, usually gelled collagen or Matrigel^®^ (Figure 3A). The ECM constitution influences cell disposition and function as the underlay matrix in sandwich cultures controls cell morphology and multicellular arrangement while the overlay matrix impacts bile excretion behavior (Deharde et al., 2016; Langhans, 2018). Sandwich-cultured hepatocytes regain polarity, maintaining proper basolateral and canalicular transporters localization and functional bile canaliculi. This 3D culture system is particularly important for the estimation of transport clearance, enzyme-transporter interplay, and bile acid mediated hepatotoxicity (Tuschl et al., 2009; Chatterjee et al., 2014; Deharde et al., 2016; Yang et al., 2016; Zeigerer et al., 2017). Data generated with sandwich models can also be used to establish quantitative relationships between intracellular bile acid accumulation and cytotoxicity and this information can be incorporated into pharmacology models for DILI and hepatic clearance predictions (Ogimura et al., 2011; Camenisch and Umehara, 2012; Umehara and Camenisch, 2012; Yang et al., 2016). Indeed, Chatterjee et al. (2014) used sandwich culture systems from hpHep and rpHep and evaluated the system with a set of compounds correctly flagging clinically known cholestatic compounds (eight out of the nine). The major limitation of sandwich cultures of hpHep is that in the long-term it has been reported leakage, bile canaliculi damage and development of cholestasis in a time-dependent manner (Deharde et al., 2016; Zeigerer et al., 2017). This indicates the need to improve culture conditions and increase the stability of the bile canalicular network necessary to model hepatobiliary excretion processes *in vitro* (Rowe et al., 2013; Deharde et al., 2016; Zeigerer et al., 2017; Bell et al., 2018). Nevertheless, sandwich cultures are a valuable tool for short-term studies of hepatobiliary drug disposition and for assessing the underlying mechanisms of hepatotoxicity.

#### Multicellular Spheroid Cultures

Three-dimensional systems of multicellular spheroids take advantage of the self-assembling capacity of cells to form aggregates and maintain cell viability, over an extended time in culture while keeping a better hepatocyte-like functional phenotype when compared to 2D cultures (Messner et al., 2013; Wrzesinski et al., 2014; Bell et al., 2016; Leite et al., 2016). The different systems for culturing multicellular spheroids are summarized in Table 2.

**TABLE 2:** Advantages and limitations of spheroid forming techniques for *in vitro* toxicity testing applications.

Spheroid forming techniques	Advantages	Limitations	References
**Non-adhesive surface Ultra-low attachment plate**	Low cost Easy to perform Co-culture of different cell types	Variation in size/cell number/shape	Leite et al., 2011, 2016; Bell et al., 2016
**Hanging drop**	Inexpensive Easy to perform Well-controlled spheroid size Fast spheroid formation Easy to trace spheroid assembly Co-culture of different cell types	Labor intensive Difficult massive production	Messner et al., 2013
**Micromolding Nanoimprinting**	Well-controlled spheroid size Designed aggregate geometry Co-culture of different cell types	High complexity Requires specialized facilities	Nakamura et al., 2011; Yoshii et al., 2011; Chan et al., 2013
**Stirred system**	Low complexity Massive production Long-term culture Dynamic control of culture conditions Adaptable to perfusion Adaptable to online monitoring Easy to scale up Co-culture of different cell types	Requires specialized equipment Requires trained personnel	Miranda et al., 2009, 2010; Leite et al., 2011
**Hydrogels/scaffolds**	Availability of a wide range of natural or synthetic materials Mimic cues of native ECM Biodegradable Protection from shear stress	Batch-to-batch variability of natural materials Requires trained personnel Labor intensive	Miranda et al., 2010; Tostoes et al., 2011; Tripathi and Melo, 2015; Christoffersson et al., 2019

Non-adhesive surfaces, hanging-drop method, hydrogels, and nanoimprinted structures are some examples of small-scale 3D systems that allow the formation of organoids or multicellular spheroids of hepatocytes (Messner et al., 2013, 2018; Bell et al., 2016; Koyama et al., 2018; Peng et al., 2018), hepatic cell lines (Ramaiahgari et al., 2014; Leite et al., 2016) or other cell types (Huch et al., 2015; Asai et al., 2017; Cipriano et al., 2017b, 2020; Takebe et al., 2017; Wang S. et al., 2019; Wang Z. et al., 2019; Ramli et al., 2020; Table 2).

The use of non-adhesive surfaces (Figure 3B) is the least complex and easier strategy to establish spheroid cultures as it does not require specialized equipment. Herein, spheroid size is controlled by the cell density, media volume and serum concentration. Using ultra-low attachment plates, Bell et al. (2016) showed that cryopreserved hpHep spheroids may constitute a promising *in vitro* system to study liver function; liver diseases such as steatosis, cholestasis, and viral hepatitis; drug targets, and delayed onset of DILI reactions since proteomic analysis of the spheroid cultures closely resembled intact liver tissues and could reflect inter-individual variability. The adequacy of the model for long-term dosing tests was also demonstrated by the higher sensitivity of 3D cultures to a panel of hepatotoxic agents (Bell et al., 2016). Moreover, spheroids of hepatic cell lines demonstrated an improved phenotype, displaying higher ALB and apolipoprotein B (ApoB) secretion and higher expression of genes related to phase I metabolism, glucose, and lipid metabolism (Nakamura et al., 2011; Takahashi et al., 2015). More recently, immortalized and expandable human liver progenitor-like cells spheroids (iHepLPCs-3D) revealed enhanced hepatic-specific functions and markers and successfully predicted individual heterogeneous toxicities of several drugs (Wang Z. et al., 2019).

Non-parenchymal cells have a key role in liver injury. Thus, the incorporation of stellate cells, Kupffer cells, and sinusoidal endothelial cells in liver cell models has been attempted for improving the prediction of drug toxicity (Messner et al., 2013; Bell et al., 2016; Leite et al., 2016; Proctor et al., 2017; Hafiz et al., 2020; Nudischer et al., 2020). Proctor et al. (2017) demonstrated the higher predictive value of 3D human liver microtissues (multicellular spheroids), consisting of a co-culture of hpHep, Kupffer cells and liver endothelial cells, due to the increased sensitivity in identifying hepatotoxic drugs within a panel of 110 compounds, when compared to 2D-cultured hpHep (Proctor et al., 2017). Spheroid co-cultures of HepaRG with human hepatic stellate cells also led to the development of a 3D *in vitro* fibrosis model, maintaining the metabolic competence of the organoid over 21 days (Leite et al., 2016). This 3D model enabled the identification of compounds that induce liver fibrosis, being suitable for repeated dosage studies and displayed differential toxicity and hepatic stellate cell activation profile according to the nature of the compound (Leite et al., 2016).

#### Dynamic Cell Culture Systems Applied to Hepatic Spheroids

A limitation of static cultures is that these types of culture do not mimic the blood flow and oxygen, nutrient, and drug gradients that occur *in vivo*. Therefore, dynamic cell culture systems have been developed to create physiologically relevant versions of such gradients (Miranda et al., 2009, 2010; Leite et al., 2011; Tostoes et al., 2011).

The NASA rotary system, a milestone in dynamic 3D culturing, is a rotating cell culture vessel that simulates a microgravity condition. The low shear force allows spheroid growth as well as high mass transfer of nutrients in media preventing cell death within the spheroid core (Brown et al., 2003). This system has been used to culture spheroids of primary hepatocytes, presenting functional bile canaliculi, up-regulation of hepatocyte-specific functional genes, glycogen storage, as well as ALB production and phase I and II enzymatic activity (Brown et al., 2003; Nelson et al., 2010; Chang and Hughes-Fulford, 2014). It also enabled culturing aggregates of iPSC-derived HLCs or of hepatic cell lines with increased up-regulation of metabolic and hepatocyte-specific gene transcripts, and expression of tight junction proteins providing a more physiologically relevant system that has even been used for the study of hepatitis viruses infections (Chang and Hughes-Fulford, 2009; Sainz et al., 2009; Berto et al., 2013; Yamashita et al., 2018). Nevertheless, translating this technology to absorption, distribution, metabolism, excretion, and toxicity (ADMET) studies has been challenging due to the expensive equipment and labor intensive loading, maintenance, and harvesting (Hammond et al., 2016).

Alternatively, the spinner flask suspension cultures (Figure 3C) are maintained in a simple and effective stirred system that has been previously described for culturing primary hepatocytes (Sakai et al., 1996; Kamihira et al., 1997; Glicklis et al., 2004; Miranda et al., 2009; Tostoes et al., 2011; Pinheiro et al., 2017), hepatic cell lines (Werner et al., 2000; Chen et al., 2014), and HLCs (Subramanian et al., 2011; Schneeberger et al., 2020). Spinner flask hepatic cultures have been used for mass production of cells for treating liver failure (Sakai et al., 1996; Kamihira et al., 1997; Schneeberger et al., 2020) and for maintaining hepatic cells for toxicological and pharmacological studies (Miranda et al., 2009; Pinheiro et al., 2017). This cell culture system offers the possibility for up-scaling (i.e., 125 mL to 36 L of working volume); adaptation to a perfusion system; online culture monitoring (Tostoes et al., 2011); and sampling of cells or cell culture medium for several analyses (Pinheiro et al., 2017), which is particularly interesting for PK studies (Miranda et al., 2009). Some studies resort to the encapsulation of hepatic cells to protect from shear stress while conferring ECM characteristics which may result in enhanced cell performance (Miranda et al., 2010; Chen et al., 2014).

By resorting to spinner flasks, primary hepatocyte spheroids (both of human and rat origin) preserved ALB and urea secretion and biotransformation activity of phase I and phase II enzymes up to 3 weeks (Miranda et al., 2009, 2010; Leite et al., 2011; Tostoes et al., 2011) and were able to metabolize diphenhydramine and troglitazone (Miranda et al., 2009); while hepatoma spheroids demonstrated gradual increase in ALB synthesis and ammonia elimination with increases in rotation speed (Chen et al., 2014). Using a similar system, Pinheiro et al. (2017) also demonstrated the maintenance of the hepatic phenotype through the presence of ALB, cytokeratin (CK)-18, HNF-4α, MRP2, and OATP-C, along with the production of urea and ALB. Stable activity levels of phase I (7-ethoxycoumarin-*O*-deethylation, ECOD, and 7-ethoxyresorufin-*O*-deethylase, EROD, activities) and phase II (sulfotransferase, SULT1A1) enzymes, modulated by nevirapine and its metabolites were also observed (Pinheiro et al., 2017). Positive results have also been obtained with co-cultures of hepatocytes and fibroblasts which demonstrates the importance of ECM interactions in hepatic phenotype (Leite et al., 2011). Moreover, spinner cultures improved *CYP3A4*, *ALB*, and *MRP2* expression in HLCs and increased ALB and urea production when compared to static cultures (Schneeberger et al., 2020) and were reported for the mass production of liver organoids presenting up-regulated hepatic markers (Subramanian et al., 2011).

#### Bioreactor Systems

Bioreactors are containers that provide the optimal requirements for biochemical reactions for the synthesis of a desired product at an industrial scale (e.g., pharmaceuticals, vaccines, or antibodies), and have been primarily developed to grow yeast, bacteria, or animal cells (Mustafa et al., 2018). Bioreactors differ from the previously mentioned dynamic systems by enabling the remote monitoring of cultures, i.e., the accurate control of cell culture parameters that may provide the appropriate stable microenvironment for liver cell cultures (Figure 3D; Tostoes et al., 2012; Lübberstedt et al., 2015; Farzaneh et al., 2020). Culture parameters include medium flow, gas tension, temperature, pH, glucose metabolism, lactate production along with the specific determination of hepatic metabolic activity revealed by ammonia detoxification, urea, and ALB secretion, enabling to extrapolate at the cell functional level. As an example, online monitoring of oxygen concentration, which is related with changes in metabolic activity, allows the estimation of cell viability in real time (Mueller et al., 2011; Rowe et al., 2018).

Bioreactors generally operate under linear or circular perfusion. The continuous addition of nutrients, mixing and removal of metabolic by-products ensures that hepatocytes experience smaller gradients of nutrients and hormones which enhance hepatocyte functionality (Tostoes et al., 2011, 2012). Accordingly, when comparing perfusion feeding with 50% medium replacement, the former showed improved ALB synthesis in non-encapsulated rpHep spheroids whilst urea synthesis and phase I drug metabolizing enzyme activity were improved in alginate encapsulated spheroids (Tostoes et al., 2011). Furthermore, the possibility of running in recirculation and feed mode allows repeated dose testing, reflecting more closely the *in vivo* situation (Mueller et al., 2011). Tostoes et al. (2012) evaluated the feasibility of using hpHep spheroids for repeated drug dose testing in an automated perfusion bioreactor for 3–4 weeks. These conditions allowed the maintenance of phase I and II enzyme expression and activity responding to induction stimuli, the presence of hepatic markers (HNF-4α, CK-18, CYP3A, and ALB) and the maintenance of ALB and urea synthesis rate. The presence of polarity markers and bile canaliculi function further supported the applicability of this system for long-term and repeated drug dose tests (Tostoes et al., 2012).

The hollow-fiber bioreactor is an example of a 3D perfused bioreactor system (Darnell et al., 2011, 2012; Mueller et al., 2011; Hoffmann et al., 2012; Freyer et al., 2016; Knospel et al., 2016; Cipriano et al., 2017b). This system was originally developed to function as extracorporeal liver support system and designed to accommodate a 3D perfusion, high-density culture of human liver cells within a cell compartment volume of 800 mL (Gerlach et al., 1994, 2003). It consists on a complex capillary network for arterio-venous medium perfusion, oxygen supply, and carbon dioxide removal, with an electronically controlled perfusion device with pumps for medium feed and recirculation, temperature control, and a valve regulated by a gas mixing unit (Darnell et al., 2011; Mueller et al., 2011). Aiming for drug testing applications, the same system was later miniaturized to cell compartment volumes of 8, 2, and 0.5 mL which enabled a significant reduction of the required cell amounts and reagents while maintaining cell function similar to larger devices (Zeilinger et al., 2011; Lübberstedt et al., 2015; Knospel et al., 2016). Human primary liver cells cultured in such small-scale hollow-fiber bioreactors preserved CYP1A2, CYP2D6, and CYP3A4/5 activities as well as the drug transporters BCRP, MDR1, and MRP2 up to 2 weeks in culture (Zeilinger et al., 2011; Hoffmann et al., 2012). Notably, these systems also displayed relevant biotransformation and toxicity profiles for several drugs, including paracetamol and diclofenac, along with the formation of biliary structures (Hoffmann et al., 2012; Lübberstedt et al., 2015; Knospel et al., 2016).

The implementation of alternative *in vitro* systems resorting to SC-derived HLCs culture in a bioreactor has also been described (Songyang et al., 2015; Freyer et al., 2016; Cipriano et al., 2017b; Farzaneh et al., 2020). Under such conditions, HLCs present glycogen storage ability, expression of hepatic-specific markers and transporters, including *CK-18, ALB, HNF-4*α, *CYP1A2, MRP2*, and *OATP-C*, formation of bile duct-like structures, higher ALB production and diclofenac biotransformation (Songyang et al., 2015; Freyer et al., 2016; Cipriano et al., 2017b). Taking advantage of the controlled microenvironment provided by bioreactors, Farzaneh et al. (2020) demonstrated the potent impact of oxygen concentration in the expression of liver-specific genes, ALB and urea secretion and CYP3A4 activity in human hepatic organoids derived from iPSCs.

#### Bioprinting

The recent emergence of 3D printer technology (bioprinting), along with the development of biocompatible materials (e.g., hydrogels), has been translated into tissue engineering, constituting a novel fabrication technique. This technology resorts to cell-laden biomaterials as bioinks (Figure 3E) and involves layer-by-layer deposition of cell-embedded polymers guided by a computer-aided design (CAD) software (Ma et al., 2018). It is considered a precise, versatile, and flexible technique that allows controlled cell patterning, thus contributing to create defined heterotypic cell contacts (Nguyen et al., 2016). It may also mimic *in vivo* ECM and, ultimately, enables to generate a functional tissue or organ. Bioprinting not only constitutes a renewed promise for regenerative and personalized medicine, with the development of patient-specific tissue designs and on-demand creation of complex structures within a short time (Aimar et al., 2019; Tamay et al., 2019), but also constitutes an opportunity for the development of the next-generation devices for toxicology and drug-screening purposes.

Currently, there are already available examples of 3D bioprinting approaches with enhanced liver cell functionalities *in vitro.* Liver organoids of HepaRG and human stellate cells printed for mimicking liver lobule presented higher *ALB* and *CYP3A4* expression than HepaRG monolayer cultures (Grix et al., 2018). Similarly, a physiologically relevant bioink allowed hpHep and liver stellate cells to maintain urea and ALB production over 2 weeks while responding to drug treatment appropriately (Mazzocchi et al., 2018). Additionally, Nguyen et al. (2016) developed human 3D bioprinted liver tissues with patient-derived hepatocytes and NPCs stable for 4 weeks in culture and identified trovafloxacin toxicity signatures at clinically relevant doses for the first time. Moreover, Kizawa et al. (2017) created a human bioprinted liver tissue maintaining stable drug, glucose metabolism and bile secretion for at least 23 days in culture.

The combination of iPSC-derived hepatic cells and bioprinting technologies has also been reported (Kazemnejad et al., 2008; Ma X. et al., 2016; Goulart et al., 2019; Yu et al., 2019). A 3D-bioprinted structure mimicking liver lobule pattern with iPSC-derived hepatic progenitor cells and human umbilical vein endothelial cells and adipose-derived SCs as supporting cells improved the expression of hepatic-specific markers, biotransformation enzymes, and ALB and urea production (Ma X. et al., 2016). Moreover, by taking advantage of the innate biochemical constituents and ultrastructure of the native ECM, Yu et al. (2019) used decellularized ECM and iPSC-derived HLCs as bioink in a hexagonal structure digitally designed, demonstrating the potential of these engineering personalized human tissue platforms.

Despite all the advances brought by 3D bioprinting, an important limitation is that this technology does not consider post-printing processes that are necessary to better mimic the *in vivo* environment such as changes in scaffold shape throughout time, resulting from, e.g., coating, cell self-organization, and matrix deposition. To address this issue, a novel technique termed “Four-dimensional (4D) bioprinting” has recently emerged in which constructs continue to evolve after being printed over time, i.e., the fourth dimension (Gao et al., 2016). Four-dimensional adds the advantages of 3D printing while using smart materials able to reshape themselves in response to different stimuli (e.g., pH, temperature, and light) to closely mimic the dynamic responses of tissues (Tamay et al., 2019). The expectation is that using 4D bioprinting technology will produce bioprinted human liver tissues containing human liver cell lines and immunocompetent cells within a defined architecture, with the aim of detecting DILI during the non-clinical phase (Poietis, 2018). Altogether, these technologies seem very promising in the quest for *in vitro* liver relevant and functional models and motivate further development for advanced pharmaceutical applications. Nevertheless, limitations such as biocompatible materials that can be printed, the inability to create microstructures and low bioprinting speeds are still some important challenges that have been hampering the possibility of running screening studies for toxicology applications (Gao et al., 2016; Mazzocchi et al., 2019; Tamay et al., 2019).

#### Microfluidic Platforms

The combination of microfabrication techniques, such as photolithography frequently used to manufacture computer chips, together with the rapid development of tissue engineering led to the establishment and expansion of systems with dimensions in the micrometer scale for cell culture purposes, i.e., the MP or organ-on-a-chip (OoC) systems (Figure 3F; Bhatia and Ingber, 2014).

Although 3D liver models allow maintenance of *in vivo*-like phenotype for several days or even weeks, the static culture conditions do not enable the removal of medium accumulated substances or metabolites, that can be toxic or introduce self-feedback inhibition of cells functionality/viability, as is the case of urea or ammonia accumulation. The need for flow-based systems granted MP or liver-on-a-chip an enormous potential, as they may recapitulate the *in vivo* flow rate by removing the metabolites and functional products. Moreover, due to its small size, the experimental costs, reagent volumes, and cell number needed within MP are lower, which is particularly interesting for high-throughput experimentation, while enabling high microenvironment control (Bhatia and Ingber, 2014; Loskill et al., 2015; Sosa-Hernández et al., 2018). Most importantly, by resorting to the microfluidic technology, it is possible to numerically define a downscaling factor of a living tissue into an *in vitro* tissue-representative functional unit that will support quantitative *in vitro* to *in vivo* extrapolations using physiologically-based modeling and PK studies, which may represent an important step toward the replacement and reduction of animal models in the non-clinical phase (Bauer et al., 2017).

Organ-on-a-chip systems display high design and experimental flexibility, offering the possibility to be planned according to the aim of the study, i.e., in a more fit-for-purpose fashion. OoC contain the minimal functional unit of a tissue, recapitulating the *in vivo* organ’s dynamics, architecture, functionality, and (patho)physiological response under real-time monitoring (Bhatia and Ingber, 2014; Mastrangeli et al., 2019), e.g., quantification of oxygen and glucose concentrations and cytokine detection (Zhou et al., 2015; Bavli et al., 2016). As such, some of the most important aspects to consider for the establishment of a OoC system are the chip design; the cell sources and cell types as well as cell density and disposition to enable the formation of functional tissues; the medium composition for each cell type; flow rate, direction, and type of perfusion; and the ability to perform functional endpoint assessment of the tissues in the chip.

Within the OoC technology, modulation of fluid flow, both in terms of direction and rate, have an impact in cells phenotype while enabling media sampling for analyses throughout culture period (Domansky et al., 2005; Wikswo, 2014; Vivares et al., 2015; Sosa-Hernández et al., 2018; Mastrangeli et al., 2019; Busche et al., 2020). MP also enable a more physiological cell-to-media ratio, avoiding dilution of signaling molecules and metabolites. High cell-to-media ratios cannot be achieved in higher scale cell culture systems without extreme costs on cell production and without compromising the maintenance of cell viability (Becker et al., 2014). Interestingly, a recent quantitative comparison on liquid-to-cell volume ratios and metabolic burden between the human body and *in vitro* systems revealed a systemic liquid-to-cell ratio of 0.3 in the human body, with 0.06 nL of liquid per hepatocyte, while the *in vitro* systems range from 375 to 0.5 depending on the scale and perfusion system (Wang et al., 2018). The functional importance of high density cell culture in low volume systems was demonstrated by Haque et al. (2016) that observed the accumulation of higher and more physiological concentrations of cytokines (which triggers autocrine signals) and increased ALB production, MRP-2 presence, bile canaliculi formation as well as CYP3A4 and 1A1 activities and *CYP1A2* expression.

The spatial arrangement of the cells is another important factor to enhance the functionality of hepatocytes, by maintaining cell polarity and tissue-specific activity (Lee et al., 2007; Kang et al., 2015; Rennert et al., 2015; Busche et al., 2020). Within MP, cells can be seeded in high densities in either a 2D or a 3D fashion (Sosa-Hernández et al., 2018). Tightly packed hepatocytes in a MP designed to simulate the liver sinusoid structure promoted properties of a functional liver sinusoid such as extensive cell–cell contact, continuous nutrient exchange and defined tissue, and fluid transport regions (Lee et al., 2007). A further advantage of these systems is that different cell types can be cultured in the same system in separate chambers, having representative cells of the same tissue or even from different organs in interaction through paracrine or endocrine chemical signals like *in vivo*, constituting multi-organ systems (Zhou et al., 2015; Liu et al., 2019). This enables the study of organ-level responses to a potential toxic compound that involve the interaction of different tissues (Ronaldson-Bouchard and Vunjak-Novakovic, 2018).

At the single organ level, Rennert et al. (2015) used a two-channel MP (Becker et al., 2014) to create a 3D liver model integrating a vascular layer, composed of endothelial cells and tissue macrophages, and a hepatic layer, comprising stellate cells co-cultured with HepaRG cells, separated by a suspended membrane simulating the space of Disse. The complexity of this model enhanced hepatocyte polarity and allowed the observation of hepatobiliary function (Rennert et al., 2015). Moreover, it incorporated a sensor for online oxygen measurement, useful for toxicological screening, as reported earlier (Rennert et al., 2015). On the other hand, Danoy et al. (2019) optimized a culture of iPSC-derived HLCs in a biochip and showed that the microfluidic environment led to a higher degree of mature HLCs than in traditional 2D cultures. In a follow-up study, the same microfluidic culture was used to mimic liver zonation based on the formation of an oxygen gradient in the biochip (Danoy et al., 2020). Moreover, when co-cultured iPSC-derived endothelial cells, iPSC-derived HLCs were able to metabolize quercetin into its active metabolites (Yu et al., 2020).

At the multi-organ level, OoC systems have been developed to recreate the first pass metabolism dynamics by connecting gut epithelial cells and liver cells (Choe et al., 2017). With these systems it is possible to consider the gut-liver axis, including immune cells to study inflammatory responses, important for diabetes and fatty liver disease models (Jeon et al., 2020). These systems can also be used to mimic an oral administration route resorting to liver–intestine co-culture (Maschmeyer et al., 2015); to mimic systemic administration routes using endothelialized liver–skin co-culture (Maschmeyer et al., 2015); and to restitute pancreas-liver functional coupling through insulin release from pancreatic islet microtissues in response to a glucose load that promoted glucose uptake by liver spheroids (Bauer et al., 2017). Finally, they can also be adequate to study drug distribution through the connection of, for example, up to ten different microphysiological systems (MPSs), including liver, gut, lung, endometrium, heart, pancreas, brain, skin, kidney, and muscle (Edington et al., 2018). In sum, MP can indeed represent a game changer for personalized medicine applications and in the development of *in vitro* non-clinical models (Wang et al., 2018; Ingber, 2020; Sohn et al., 2020).

### *In vitro* Hepatotoxicity Studies: 3D Versus 2D

The evaluation of the capacity of a given model system to detect and mimic prototypical types of liver toxicity allows to determine its ultimate predictive ability. This toxicity must be assessed both in a short-term (24–48 h) and a long-term (weeks to months) culture conditions as it is known that DILI mechanisms may be developed not only after an acute exposure but also from a chronic type (e.g., by development of drug tolerance or deposition of elimination products) (Jiang et al., 2019). Moreover, the assessment of a prolonged exposure to each compound leads to substantially lower IC_50_ values obtained in standard cytotoxicity assays, representing a cumulative effect often seen in the clinic and not only in an overdose scheme following isolated supratherapeutic administrations. Therefore, for a more complete evaluation of the strategies used in different studies, it is important to gather the existing data regarding well-known hepatotoxicants. In this work, we selected three of the *most DILI-concern* medicines, ranked by the United States Food and Drug Administration, based on their impact in human health, either because they are indeed highly prescribed (diclofenac and paracetamol) or are paradigmatic examples in Toxicology (troglitazone) (Chen et al., 2020). In fact, molecules such as paracetamol or diclofenac display a well-known diversity of mechanisms of hepatotoxicity that may help to further validate new *in vitro* liver models. Several hepatotoxicity studies that evaluate paracetamol (Supplementary Table 1), diclofenac (Supplementary Table 2), and troglitazone (Supplementary Table 3) consider distinct cell sources, culture conditions, and endpoints. However, the available data is not homogeneous and thus it is somehow difficult to compare results between groups, especially considering 3D liver models. Indeed, full characterization of the *in vitro* liver models regarding metabolic and toxicity capacity is not always described, which may represent one of the reasons limiting their acceptance by the regulatory authorities and further effective application in toxicological studies.

As shown in Supplementary Tables 1–3, toxicities of test drugs are often investigated by evaluation of cytotoxicity biomarkers such as cell viability (e.g., tetrazolium reduction MTT or MTS assays), membrane lysis (e.g., LDH release) or depletion of cellular ATP. However, these represent only late-stage toxicity associated with apoptotic or necrotic events and thus do not permit a proper mechanistic evaluation of the toxicological events (O’Brien et al., 2006). Therefore, additional mechanistic endpoints have gained increasing importance when assessing drug safety with the pharmaceutical industry and the scientific community proposing complementary biomarkers’ assessment, in order to obtain and cover different mechanisms of injury to diminish hepatotoxicity risk. These include mitochondrial dysfunction, bile salt transporter modification, lipids accumulation, reactive metabolite formation through conjugation with GSH or covalent binding and calcium homeostasis alteration that need to be assessed in a representative number of compounds with different toxicity mechanisms within high-content and high-throughput platforms (Xu et al., 2004; O’Brien et al., 2006; Khetani et al., 2013; Trask et al., 2014; Schadt et al., 2015; Bell et al., 2016; Williams et al., 2020; Zhang C. et al., 2020). Hence, besides including the different hepatic cell source and cell culture systems, Supplementary Tables 1–3 were incorporated to not only accommodate the cytotoxicity data but also the metabolic activity and mechanistic endpoints assessed in each study. The few studies that evaluate these mechanistic biomarkers are essential to identify also the models’ ability to mimic processes related to cholestasis, steatosis, genotoxicity, and viral hepatitis (Shen et al., 2012; Bell et al., 2016; Hendriks et al., 2016; Leite et al., 2016; Prill et al., 2016; Williams et al., 2020), amongst others.

#### Paracetamol

Paracetamol is a widely used antipyretic and non-opioid analgesic agent that constitutes an example of a safe drug at therapeutic doses, but overdosage causes predictable and reproducible hepatotoxicity through mitochondrial dysfunction and centrilobular necrosis in the liver (Hinson et al., 2010).

Paracetamol is metabolized mainly by conjugation with sulfate and glucuronic acid (Riches et al., 2009) and, in a less extent, by oxidation by CY2E1, CYP1A2, CYP2D6, CYP2A6, and CYP3A4 (Mazaleuskaya et al., 2015). As previously stated, its oxidation generates NAPQI that is detoxified by GSH conjugation, through glutathione S-transferases (GSTs) GSTP1, GSTT1, and GSTM1. When large quantities of NAPQI are formed, liver GSH pool can be critically depleted, meaning that excess NAPQI is not detoxified and cell injury occurs, namely trough the modification of cellular proteins. Protein binding leads to oxidative stress and mitochondrial damage (McGill and Jaeschke, 2013; Caparrotta et al., 2018). Paracetamol toxicity is also related to calcium accumulation and activation of endonucleases, DNA damage (Boelsterli, 2003), ATP depletion, *Jnk* activation, up-regulation of electron transport chain protein components and activation of *p53* signaling (Davis and Stamper, 2016).

Supplementary Table 1 summarizes the collected *in vitro* data for paracetamol. It suggests that mouse primary hepatocytes are more sensitive to paracetamol, with lower IC_50_ values (Jemnitz et al., 2008; Kučera et al., 2017), followed by rpHep, hepatic cell lines HepG2 and HepaRG, hpHep and HLCs (Lewerenz et al., 2003; Jemnitz et al., 2008; Riches et al., 2009; Zhang et al., 2011; Tasnim et al., 2015; Bell et al., 2017), highlighting not only the interspecies differences but also the importance of choosing a representative cell type (Carmo et al., 2004; Reder-Hilz et al., 2004).

Paracetamol IC_50_ values compiled in Supplementary Table 1 further suggest that 2D cultures are less sensitive to paracetamol toxicity than 3D cultures (Gunness et al., 2013; Jang et al., 2015; Gaskell et al., 2016; Bell et al., 2017; Li et al., 2020). It was demonstrated increased sensitivity of 3D human liver microtissues, with subsequently lower IC_50_ values for a panel of known hepatotoxicants, including paracetamol, in comparison with 2D-plated hpHep (Proctor et al., 2017). Moreover, by using a 3D liver-sinusoid-on-a-chip of HepG2, Deng et al. (2019) showed that not only this system was able to improve cell functions, but also its sensitivity to paracetamol when compared to 2D-plated HepG2. Besides, even within 3D systems, different culture strategies may lead to different results for the same drug. Jang et al. (2015) tested HepG2 in a 3D static Matrigel^®^ culture and in a 3D microfluidic chip and obtained a higher sensitivity for hepatotoxicity in the latter, justified by its improved maintenance of hepatic functions. Foster et al. (2019) found also an increased sensitivity in 3D co-culture systems of hpHep and NPCs compared to hpHep spheroids. Likewise, Li et al. (2020) observed an augmented toxicity to paracetamol in the co-culture spheroids group (hpHep and Kupffer cells). Interestingly, when both systems were co-treated with lipopolysaccharides (mimicking inflammatory conditions), a higher protective role was detected in the co-culture system, mainly due to Kupffer cells, when comparing to hpHep spheroids. In fact, exposure and response to paracetamol under healthy or pre-inflammatory states may lead to different cytokine release profiles with distinct activation of immune cells (Kim et al., 2017; Li et al., 2020). Although it is generally acknowledged that paracetamol has only very weak anti-inflammatory properties, it should be highlighted that is commonly administered already under an inflammatory condition. This reinforces the need of considering co-cultures for an early identification of possible drug-induced hepatotoxic immunological responses depending on the patient health state.

Although mechanistic endpoints are not often represented in the majority of studies using paracetamol as hepatotoxicant, the reports that present this important information assess mainly mitochondrial dysfunction and oxidative stress (Bruderer et al., 2015; Goda et al., 2016; Zhang C. et al., 2020), followed by reactive metabolites formation and liver cholestasis or steatosis (Lewerenz et al., 2003; Prot et al., 2012; Prill et al., 2016; Kučera et al., 2017; Williams et al., 2020) and liver fibrosis (Leite et al., 2016). Nevertheless, the drastic differences in sensitivity to paracetamol, may be due not only to the cell type but also to the different culture conditions, highlighting the importance of both the cell architecture and the presence of other liver cell types for the study of distinct pathways that may be involved in drug toxicity.

#### Diclofenac

Diclofenac is one of the most worldwide prescribed NSAID. It has been linked with rare, albeit significant, cases of severe hepatotoxicity with a fatality rate of 10% (Aithal, 2004). Diclofenac is mainly metabolized by CYP2C9 into 4-OH-diclofenac and in a lower extent converted into 5-OH-diclofenac by CYP3A4. Diclofenac and its metabolites are conjugated with glucuronic acid by UGT2B7 and excreted across the canalicular plasma membrane into the bile via MRP-2 (Aithal, 2004). There is evidence that individuals that present increased glucuronidation activity as a consequence of a genetic polymorphism in UGT2B7 (C-161T allele) exhibit a 9-fold increased risk of adverse hepatic reactions (Daly et al., 2007). Diclofenac-acyl-glucuronide may also conjugate with GSH forming a diclofenac glutathione thioester (Syed et al., 2016). Thus, diclofenac metabolism comprises phase I, II, and III activities and its toxic effects can be associated to the reactive metabolites 4-OH-diclofenac, diclofenac-acyl-glucuronide, diclofenac glutathione thioester, and 4-OH-diclofenac-acyl-glucuronide (Aithal, 2004) that cause ATP depletion resulting in mitochondrial toxicity (Syed et al., 2016). As such, diclofenac constitutes an example of a hepatotoxicant dependent on bioactivation.

Compared to paracetamol, the available studies of diclofenac addressing the toxicity in 2D versus 3D cell cultures are fewer (Supplementary Table 2). Most importantly, regarding mechanistic biomarkers of toxicity, only a minority of studies assess endpoints for mitochondrial dysfunction (Goda et al., 2016), reactive metabolite formation, calcium homeostasis (Ponsoda et al., 1995), and liver cholestasis or steatosis (Bell et al., 2016; Williams et al., 2020).

As shown in Supplementary Table 2, Gaskell et al. (2016) and Cipriano et al. (2017b) obtained IC_50_ values for the 3D spheroid cultures of HepG2/C3A and HLCs, respectively, lower than for the corresponding 2D cultures. This may be due to an increase in phase II (glucuronidation) activity, indicating that the 3D system may be more representative of the biological response. Ramaiahgari et al. (2014) attained higher sensitivity to diclofenac in the 3D culture of HepG2 compared to 2D but the IC_50_ values were higher than those reported by Gaskell et al. (2016). Yu K. N. et al. (2018) also demonstrated that the addition of both phase I and II enzymes in a 3D miniaturized Hep3B cell system led to a more predictive assessment of diclofenac’s toxicity when compared to the 3D system groups with only CYP450 enzymes or human liver microsomes added and its 2D counterpart. Also, Atienzar et al. (2014) proved that the co-culture of dog hepatocytes with NPCs present similar sensitivity to diclofenac compared to HepG2 but less sensitivity than hpHep in a 5-day exposure culture. This IC_50_ variation is transversal to the majority of reports presented in Supplementary Table 2, in which most of the studies only evaluate one type of culture system (Wang et al., 2002; Xu et al., 2003; Lauer et al., 2009; Lin et al., 2012; Goda et al., 2016; Knospel et al., 2016; Sarkar et al., 2017), hindering the comparison between both types of cultures. Besides the relevance of the culture system, this observation reinforces the importance to include mechanistic insights in early toxicity assessments rather than rely solely on cell viability quantification. A competent and complete *in vitro* model may provide a higher amount of valuable information if more variables are taken into account.

#### Troglitazone

Troglitazone (TGZ) is a thiazolidinedione derivative developed for the treatment of type II diabetes. Soon after being approved, TGZ was withdrawn from the marked in Europe and 3 years afterward in the United States due to non-immune idiosyncratic toxicity (Chojkier, 2005). It is a classic example of a drug whose toxicity failed to be predicted during drug development. TGZ is extensively metabolized by CYP3A4 and GST, it is a CYP3A and 2B6 inducer and is able, as well as its sulfate conjugate, to inhibit BSEP (Sahi et al., 2000; Kassahun et al., 2001). This leads to an increased formation of TGZ metabolites along with its intracellular accumulation, resulting in intrahepatic cholestasis, mitochondrial dysfunction, covalent binding to proteins, and macromolecular damage ultimately leading to apoptosis (Smith, 2003; Chojkier, 2005). TGZ is more toxic in humans than in rodent models (Shen, 2007; Kostadinova et al., 2013). In view of this, TGZ was only identified as hepatotoxic after reaching the market. TGZ is, thus, an example of the importance of identifying species-specific toxicity. In fact, Shen et al. (2012), using 3D gel entrapped rat and human hepatocytes, observed that at clinical doses of TGZ, hepatotoxicity was absent in rat hepatocytes, but present in human hepatocytes. Similarly, Kostadinova et al. (2013) reported TGZ-induced cytotoxicity in human 3D liver cells but only minor effects in rat 3D liver.

In addition, as summarized in Supplementary Table 3, and in accordance with clinical observations, 3D human liver models show increased sensitivity to TGZ toxicity which may be related to higher formation of metabolites as consequence of the higher induction ability of 3D models. Nonetheless, Gunness et al. (2013) obtained discrepant results, with the 3D model of HepaRG cells being 10-fold less sensitive to TGZ than the monolayer model. On the other hand, this decreased sensitivity might reflect a higher clearance, commonly increased in 3D cultures due to the cellular architecture. Moreover, HepaRG cells present higher CYP3A4 activity (Aninat et al., 2006) that might interfere with drug and metabolite clearances since TGZ metabolites may also be hepatotoxic (Tolosa et al., 2018). Another explanation could be the known TGZ-induced BSEP inhibition (Jackson et al., 2018), not investigated in this study. Hendriks et al. (2016) assessed BSEP inhibition in two long-term 3D spheroid models of HepaRG and hpHep with repeated drug exposure and bile acids co-exposure and was able to detect the cholestatic effect of several compounds, including TGZ, in both cell types. Independently of the studied mechanisms, the co-culture of hpHep (Kostadinova et al., 2013; Proctor et al., 2017; Li et al., 2020) or hepatoma cell lines (Granitzny et al., 2017) with NPCs displayed a higher sensitivity to TGZ toxicity (Kostadinova et al., 2013).

Using HLCs as alternative sources to hpHep, Tasnim et al. (2016) found similar sensitivity between 3D hESC/hiPSC-HLCs and hpHep upon exposure to TGZ for 24 h, whereas when comparing to its 2D counterpart, 3D cellulosic scaffold cultured hiPSC-HLCs showed a slightly lower sensitivity. On the other hand, Takayama et al. (2013) showed that 3D-cultured iPSC-HLCs presented a decreased sensitivity to TGZ after 24 h of exposure when compared to hpHep, but a better sensitivity compared to 3D-cultured HepG2. Moreover, Holmgren et al. (2014) was able to use hiPSC-HLCs in a long-term culture and successfully detected steatosis in the cells exposed to TGZ for 2 days. This is an important step to show that these HLCs may reveal the mechanistic pattern of TGZ hepatotoxicity and stand as a good alternative for hpHep.

## Challenges Within the Assessment of Drugs Hepatotoxicity Using *in vitro* Liver Models

The pharmaceutical industry is clearly interested on the early identification of toxicity cues in models covering different aspects of human liver (patho)physiology (Bale et al., 2014). On the other hand, the chemical/cosmetic industry has been politically stressed to use advanced alternatives for animal testing for hazard identification and characterization. Although each type of industry presents different needs and goals, the early identification of potential hepatotoxic substances and deep understanding of hepatotoxic mechanisms in relevant *in vitro* models will profit both industries.

**FIGURE 4:**
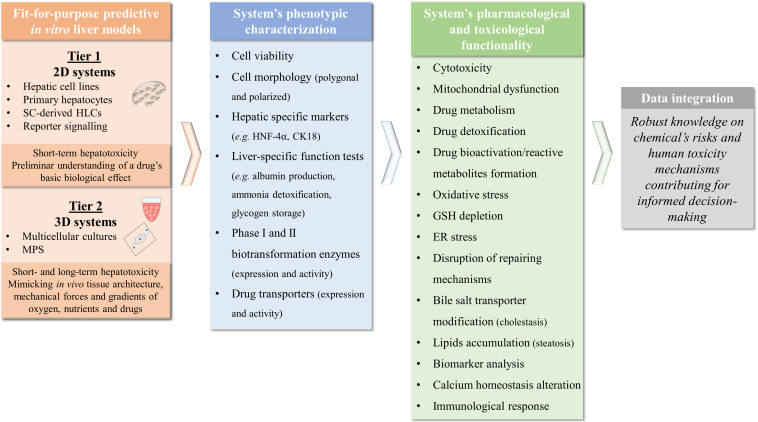
Roadmap for assessing drugs hepatotoxicity mechanisms using *in vitro* models that might be used alone or in combination at different points and on different scales. Tier 1 comprises single-cell systems that report on immediate chemical/biological effects such as cytotoxicity and bioactivation while Tier 2 includes more complex systems containing liver cells in a more physiologic state, enabling assessment of the consequences of acute and chronic drug exposure. Moreover, phenotypic characterization and the pharmacological and toxicological functionality of a system and the ability to identify toxicity mechanisms needs to be considered before undertaking toxicological investigations to ensure that the most appropriate methods are used. Depending on the complexity, each model might be able to represent one or more liver functional endpoints and can be used alone or in combination depending on the hepatotoxicity mechanisms that are intended to study. To integrate findings from different test systems and to dissect the multilevel impact of compounds, bioinformatics and machine learning models may also be useful, which will ultimately contribute to more informed decision-making in the drug development and risk assessment fields. SC, stem cells; HLCs, hepatocyte-like cells; MPS, microphysiological system.

**FIGURE 5:**
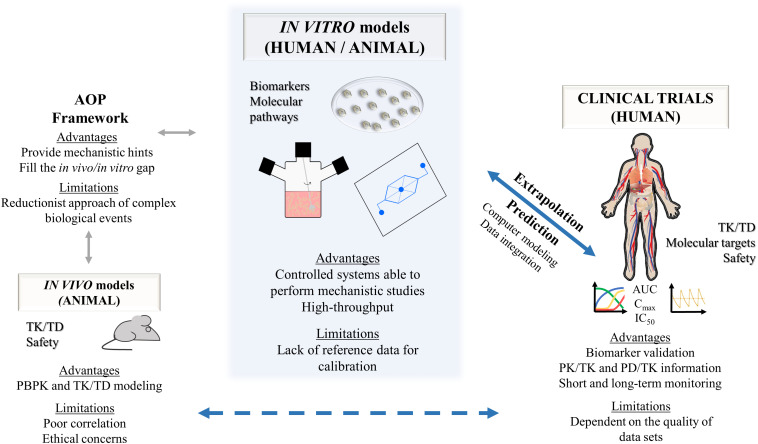
Data integration from non-clinical assays for prediction of clinical conditions. A shift in paradigm where fit-for-purpose human-based *in vitro* models, particularly using *in vitro* 3D systems and causality-inferring bioinformatic approaches, might provide high-quality data for relevant extrapolation of human toxicokinetics and toxicodynamics, ultimately leading to the prediction of human hepatotoxicity mechanisms and molecules’ risk assessment. As a consequence, animal models will then become progressively less used with the increasing complexity and relevance of these strategies. AOP, adverse outcome pathway; AUC, area under the curve; C_*max*_, maximum plasma concentration; IC_50_, half maximal inhibitory concentration; PBPK, physiologically based pharmacokinetic; PK, pharmacokinetic; PD, pharmacodynamic; TD, toxicodynamics; TK, toxicokinetics.

In this section, we propose a roadmap with the essential steps to assess drugs hepatotoxicity using *in vitro* liver models. As no single currently used model can recapitulate all human hepatotoxicity mechanisms, we further support the need for a systematic tiered approach for drug hepatotoxicity assessment combining more than one model. Indeed, the model systems with increased biological complexity might be efficiently and effectively used alone or in combination, at different points, and on different scales of the drug development process. The final goal is thus to cover the different human hepatotoxicity mechanisms needed to provide accurate hazard identification and risk assessment (Figure 4). Finally, the data generated in such non-clinical assays should be integrated to deliver robust information to drug regulators to improve the decision-making process (Figures 4, 5).

Firstly, the cell source needs to be carefully considered to ensure the appropriate tissue context. Hepatotoxicity involves different mechanisms in a multistep and multicellular process (Grattagliano et al., 2009; Gregus, 2013). As such, there is no single model or test that can evaluate a chemical’s risk of inducing liver injury, but rather a set of well-characterized hepatic models with well-defined purposes to be used in a multistep manner. This approach may vary in terms of cell source and culture complexity, allowing to assess specific toxicity mechanisms as well as to properly mimic the different types and stages of liver toxicity.

In particular, human-derived cells, namely hpHep or SC-derived HLCs, contribute to more relevant *in vitro* systems by allowing to capture population heterogeneity and to represent healthy and disease conditions. On the other hand, simpler and single-cell system based on, e.g., hepatic cell lines and monolayer cultures enable high-throughput applications for testing a wide set of conditions and allow to preliminarily understand a drug’s basic biological effects in human cells (Figure 4, tier 1). Conversely, more complex systems, such as 3D-based cultures improve data accuracy not only concerning cytotoxicity, but also biotransformation activity and drug accumulation by mimicking tissue architecture, mechanical forces and gradients of oxygen, nutrients, and drugs that are found *in vivo* (Figure 4, tier 2). Additionally, complex models that include different liver cell types, e.g., hepatocytes and stellate cells or immune cells, allow to explore additional biological responses such as cholestasis, steatosis, fibrosis, and inflammation (Leite et al., 2016; Jeon et al., 2020).

A crucial step within the development and selection of a relevant *in vitro* model for non-clinical studies is its thorough characterization. The systems’ phenotype and functionality need to be assessed to understand which pharmacology and toxicology mechanisms can be accurately represented and evaluated in each model. Importantly, the capacity of the models for both short and/or long-term exposures should be evaluated as well (Figure 4). Indeed, the models’ comparison presented in section “*In vitro* Hepatotoxicity Studies: 3D Versus 2D” revealed to be challenging and little informative because most of the hepatotoxicity studies available in the literature focus on cytotoxicity estimation instead of analyzing specific functional endpoints of hepatotoxicity such as altered conversion of primary and secondary metabolites, disruption of repairing mechanisms, immunological response, mitochondrial dysfunction, bile salt transporter modification, lipids accumulation, or calcium homeostasis alteration, which highlight hepatic injury mechanisms such as inflammation, cholestasis, steatosis, fibrosis, or genotoxicity (Xu et al., 2004; O’Brien et al., 2006; Khetani et al., 2013; Trask et al., 2014; Schadt et al., 2015; Bell et al., 2016; Leite et al., 2016; Jeon et al., 2020; Williams et al., 2020; Zhang C. et al., 2020).

Retrospective analysis of approved or failed drugs with a known mechanism displaying the expected hepatic response is a common approach to determine the capability of an *in vitro* system to accurately recapitulate the human liver biological response (Jemnitz et al., 2008; Kostadinova et al., 2013; Messner et al., 2013; Hendriks et al., 2016; Bell et al., 2017; Cipriano et al., 2017b; Proctor et al., 2017; Sarkar et al., 2017; Foster et al., 2019; Williams et al., 2020), e.g., paracetamol for induced necrosis, valproic acid for induced steatosis, or cyclosporine A for induced cholestasis (Vinken and Hengstler, 2018). The data generated with these systems should then be compared with clinical data as well as with other *in vitro* methods to understand applicability and limitations of each system for assessing the inherent risk of a chemical or a new molecular entity (Beken et al., 2016; Figure 5). Models are commonly assessed by direct comparison of *in vitro* to the *in vivo* maximum plasma concentration (C_*max*_) (Godoy et al., 2013; Shah et al., 2015; Proctor et al., 2017), often multiplied by a factor of 20× to 100× (O’Brien et al., 2006; Xu et al., 2008; Khetani et al., 2013; Proctor et al., 2017; Vorrink et al., 2018; Yu K. N. et al., 2018). Thus, knowing that the C_*max*_ of paracetamol, diclofenac, and TGZ is 165.38 μM, 10.13 μM (Regenthal et al., 1999) and 2.82 μM (Loi et al., 1999), respectively, it is possible to comprehend by Supplementary Tables 1–3 that, in general, 3D models present better capability of predicting cytotoxicity than 2D models. However, this comparison assumes that the ratio of test compounds in blood or plasma *in vivo* is the same in the cell culture medium *in vitro* (Vinken and Hengstler, 2018) and that cells response *in vivo* is the same *in vitro*. Also, within the same drug, such a big range of concentration (20× to 100× C_*max*_) may represent different mechanisms of toxicity (Albrecht et al., 2019).

A paradigm change from selecting a list of reference chemicals that cause cytotoxicity, to selecting a group of positive and negative mechanistic controls, coupled to a mechanistic-based selection of functional endpoints, including biomarkers assessment, is thus essential for using advanced *in vitro* models to its full potential. An example of a mechanistic positive control for a test system where biliary excretion is measured would be TGZ since it is a known BSEP inhibitor associated with bile acid accumulation (Funk et al., 2001; Jackson et al., 2018). Moreover, the combination of advanced 3D hepatic *in vitro* models with other advanced endpoint methodologies and systems biology employing -*omics* approaches (e.g., RNA-seq, Epigenetics, and ChIP-seq proteomics) could further support better prediction of hepatotoxicity.

Finally, the integration of the biological data obtained with the selected *in vitro* models may support a deeper understanding of the models’ potential to predict specific mechanisms of detoxification and toxicity. The inclusion of relevant positive and negative controls that validate the obtained data will support robust knowledge on chemical’s risks and human toxicity mechanisms that has not been detected so far using monolayers of a single cell type.

## Conclusion

There has been major progress toward the development of more physiologically relevant hepatic *in vitro* models, namely through the application of 3D culture techniques. Despite the wide variety of available cellular models along with distinct cell sources, 3D-based liver systems and co-culture strategies improve hepatic-specific functions and sustain the culture through longer periods than 2D counterparts, emphasizing the value-added capacity of such systems to mimic the multicellular mechanisms involved both in intrinsic and idiosyncratic liver toxicity. This has been demonstrated in paracetamol, diclofenac and TGZ toxicities studies where IC_50_ values from 3D cultures confirmed its higher sensitivity.

Nevertheless, the appropriate *in vitro* system’s evaluation and proper extrapolation of the *in vitro* data requires a paradigm shift from quantitative cytotoxicity assessment to a comprehensive mechanistic evaluation of the response to chemicals. Advanced 3D systems provide the opportunity to investigate mechanistic hepatotoxicology if accompanied by a careful selection of adequate positive and negative controls and systems toxicology data coupled to disease-related functional endpoints. Despite the advances in creating more physiologically relevant culture systems, a major difficulty is still the standardization of protocols across laboratories and the selection of critical positive and negative controls to assess the transferability and reproducibility of models across different groups. Importantly, advanced endpoint methodologies are essential to identify the applicability of each hepatic system, further allowing comparison between studies. Such a detailed and complete *in vitro* evaluation will support future decision on the adequate model to be used in chemical risk assessment and in a non-clinical drug development scheme.

## Author Contributions

JM, MC, JR, and AS: conception and design. JM, MC, JR, AS, AR, and NO: writing and critically review the manuscript. JR, AS, and AR: figure design and elaboration. JM: directing manuscript. All authors contributed to the article and approved the submitted version.

## Conflict of Interest

The authors declare that the research was conducted in the absence of any commercial or financial relationships that could be construed as a potential conflict of interest.
