# Supplementary material for: A Critical Perspective on 3D Liver Models for Drug Metabolism and Toxicology Studies
Source: Front Cell Dev Biol. 2021 Feb 22;9:626805. doi: 10.3389/fcell.2021.626805 (PMC7957963; doi:10.3389/fcell.2021.626805)
Supplement: Supplementary file 1 [file Table_1.docx]

Table S1. Paracetamol cytotoxicity, metabolism and mechanistic endpoints evaluation in different cell types and cell culture systems.

| **Cell Type** | **Cell Culture System** | **Time of Exposure** | **IC_50_ /EC_50_ / LC_50_ / TC_50_ (mM)** | **Cytotoxicity Endpoints** | **Biotransformation \| Mechanistic Endpoints** | **References** |
| --- | --- | --- | --- | --- | --- | --- |
| HepG2 | 2D | 24h | 29.7 | WST-1 assay | NA | (Wang et al., 2002) |
|  |  |  | 24.0 | ATP quantification | CYP activity, glucuronidation and sulfation activity, hepatobiliary transport \| NA | (Ramaiahgari et al., 2014) |
|  |  |  | 23.8 | Calcein-AM (live) / ethidium homodimer (dead) fluorescence | CYP1A1/2 and glucuronidation activity, drug-drug interaction \| NA | (Deng et al., 2019) |
|  |  | 72h | 15.8 |  | CYP1A1/2 induction \| NA | (Jang et al., 2015) |
|  |  | 5 days (compound addition at D0 and 2) | 2.251 | Live cell protease activity | CYP activity, glucuronidation and sulfation activity \| NA | (Atienzar et al., 2014) |
|  | 3D Spheroids with Matrigel | 6 days (repeated exposure) | 9.4 | ATP quantification | CYP activity, glucuronidation and sulfation activity, hepatobiliary transport \| NA | (Ramaiahgari et al., 2014) |
|  |  | 72h | 11.8 | Calcein-AM (live) / ethidium homodimer (dead) fluorescence | CYP1A1/2 induction \| NA | (Jang et al., 2015) |
|  | 3D Spheroids in a microfluidic chip – OrganoPlate^TM^ (MIMETAS) |  | 7.1 |  |  |  |
|  | 3D liver-sinusoid-on-a-chip (LSOC) | 24h | 9.8 |  | CYP1A1/2 and glucuronidation activity, drug-drug interaction \| NA | (Deng et al., 2019) |
| HepG2/C3A | 2D | 24h | 33.8 | ATP Quantification | CYP2E1 expression and transporter function \| NA | (Gaskell et al., 2016) |
|  | 3D Spheroids | 24h after 21 days in culture | 40.0 | ATP Quantification | NA | (Fey and Wrzesinski, 2012) |
|  | 3D Spheroids on Agarose overlay | 24h | 7.2 |  | CYP2E1 expression and transporter function \| NA | (Gaskell et al., 2016) |
|  | 3D Spheroids on a perfused microbioreactor | 12h | 12.3 | TUNEL assay | NA \| Mitochondrial function and metabolic activity (oxygen uptake), phospholipidosis and microvesicular steatosis induction | (Prill et al., 2016) |
| Hep3B | 2D | 72h | 0.3 | MTT Assay | NA | (Yu et al., 2018) |
|  | 3D Miniaturized cell-culture array (DataChip + Metachip) | 24h | 1.200 | Calcein-AM (live) / ethidium homodimer- (dead) fluorescence |  |  |
|  | 3D Miniaturized cell-culture array (DataChip) + CYP450 enzymes (MetaChip) |  | 0.068 |  |  |  |
|  | 3D Miniaturized cell-culture array (DataChip) + CYP450 + phase II enzymes (MetaChip) |  | > 1.200 |  |  |  |
|  | 3D Miniaturized cell-culture array (DataChip) + human liver microsomes (MetaChip) |  | >1.200 |  |  |  |
| HepaRG | 2D | 24h | Toxicity starting on 4.0 mM* | Alamar Blue | Metabolic competence and metabolite quantification \| Mitochondrial ROS levels and MMP measurement | (Zhang et al., 2020) |
|  |  | 24h after 5 days in culture  24h after 22 days in culture | 26.3  34.6 | ATP Quantification | CYP2E1 and MRP-2 activity \| NA | (Gunness et al., 2013) |
|  |  | 48h  7 days  14 days | 5.916  1.587  1.311 |  | *CYP, GSTT1, UGT1A1, ABCB11, ABCC1* and *SLCO1B1* gene expression \| NA | (Bell et al., 2017) |
|  | 3D Spheroids | 24h after 5 days in culture  24h after 22 days in culture | 2.7  10.1 | ATP Quantification | CYP2E1 and MRP-2 activity \| NA | (Gunness et al., 2013) |
|  |  | 24h after 6/7 days in culture  24h after 21/22 days in culture | 11.6 |  | CYP induction \| mitochondrial function (OCR) and glycolytic activity (ECAR), fibrosis (HSC activation, collagen secretion and deposition) | (Leite et al., 2016) |
|  | 3D Spheroids with pulverized liver biomatrix scaffolds | 24h | 20.0  (toxicity starting on 0.8 mM*) | Alamar Blue | Metabolic competence and metabolite quantification \| mitochondrial ROS levels and MMP measurement | (Zhang et al., 2020) |
| HLCs  (hESC) | 2D | 24h | 46.0 | MTT Assay | CYP activity \| NA | (Tasnim et al., 2015) |
| HLCs  (hiPSC) | 2D | 48h  7 days  14 days | >10.0  >10.0  9.439 | ATP Quantification | *CYP, GSTT1, UGT1A1, ABCB11, ABCC1* and *SLCO1B1* gene expression \| NA | (Bell et al., 2017) |
| rpHep | 2D | 12h | 30.0 | MTT assay | NA \| Oxidative stress, lipid peroxidation (MDA release), DNA strand breaks | (Lewerenz et al., 2003) |
|  |  | 24h | 7.6 |  | CYP2E1 activity \| NA | (Jemnitz et al., 2008) |
|  |  |  | 14.0 | WST-1 assay | NA | (Wang et al., 2002) |
|  |  |  | ~3.75 | LDH Leakage | NA \| Intracellular GSH,  ROS and MDA production, MMP visualization | (Kučera et al., 2017) |
|  |  |  | 30.0 | Calcein AM (live) / PI (dead) fluorescent stain | CYP activity \| NA | (Zhang et al., 2011) |
|  | 3D Scaffold perfused bioreactor (RoboTox) | 24h | 7.0 | Calcein AM (live) / PI (dead) fluorescent stain |  |  |
| mpHep | 2D | 24h | ~1.25 | LDH Leakage | NA \| Intracellular GSH,  ROS and MDA production, MMP visualization | (Kučera et al., 2017) |
|  | 3D Collagen sandwich | 24h | 3.8 | MTT assay | CYP2E1 activity \| NA | (Jemnitz et al., 2008) |
| hpHep | 3D Collagen sandwich | 24h | 28.2 | MTT assay | CYP2E1 activity \| NA | (Jemnitz et al., 2008) |
| Cryo hpHep | 2D | 24h | 5-10 | Live cell protease /caspase-3/7 | NA \| Mitochondrial dysfunction (OCR) | (Goda et al., 2016) |
|  |  |  | 45.2 | MTT Assay | CYP activity \| NA | (Tasnim et al., 2015) |
|  |  |  | ~20.0 | ATP Quantification | Sulfation and  glucuronidation assessment \| NA | (Riches et al., 2009) |
|  |  |  | >20.0 |  | NA \| inflammatory response | (Li et al., 2020) |
|  |  | 48h | 4.596 |  | NA \| miR-122, HMGB1 and α-GST | (Proctor et al., 2017) |
|  |  | 5 days (compound addition at D0 and 2) | 2.987 | Live cell protease activity | CYP activity, glucuronidation and sulfation activity \| NA | (Atienzar et al., 2014) |
|  | 3D Spheroids | 48h  7 days  14 days | >10.0  2.703  0.644 | ATP Quantification | *CYP, GSTT1, UGT1A1, ABCB11, ABCC1* and *OATP-C* gene expression \| NA | (Bell et al., 2017) |
|  |  | 24h after repeated dosing at D8, 12 and 15 | 3.280 |  | NA \| inflammatory response | (Li et al., 2020) |
| Co-culture of HepaRG and  HSC | 3D Spheroids | 24h after 6/7 days in culture  24h after 21/22 days in culture | 10.0  8.0 | ATP Quantification | CYP induction \| mitochondrial function (OCR) and glycolytic activity (ECAR), fibrosis (HSC activation, collagen secretion and deposition) | (Leite et al., 2016) |
| Co-culture of dog hepatocytes and NPC | 2D | 5 days (compound addition at D0 and 2) | 8.489 | Live cell protease activity | CYP activity, glucuronidation and sulfation activity \| NA | (Atienzar et al., 2014) |
| Co-culture of cryo hpHep and NPC | 3D Spheroids | 24h  72h  7 days  10 days | >10  2.9  1.9  1.7 | ATP Quantification | Metabolite quantification \| miR-122, α-GST | (Foster et al., 2019) |
|  | 3D Spheroid human liver microtissues (3D hLiMT) | 72h | 1.348 |  | Biotransformation phase I, II and III enzymes induction \| Mitochondrial  oxidative stress, NAPQI-protein adducts quantification | (Bruderer et al., 2015) |
|  |  | 5-6 days  14 days | 0.9110  0.5728 |  | NA \| miR-122, HMGB1 and α-GST | (Proctor et al., 2017) |
|  |  | 14 days | 0.7542 |  | NA | (Messner et al., 2013) |
| Co-culture of cryo hpHep and KC | 3D Spheroids | 5 days | 2.247 | ATP Quantification | NA \| inflammatory response | (Li et al., 2020) |
| Co-culture of cryo hpHep and LSEC | 3D Human Liver-Chip model | 10 days | 2.4 | ATP Quantification | Metabolite quantification \| miR-122, α-GST | (Foster et al., 2019) |

ABC, ATP Binding Cassette; Ca^2+^, calcium; cryo, cryopreserved; CYP, cytochrome P450; ECAR, extracellular acidification rates; GSH, glutathione; GST, glutathione S-transferase; HepG2, HepG2/C3A, FaO, Huh7, HCCT-T, hepatic cell lines; hESC, human embryonic stem cells; HLC, hepatocyte-like cells (stem cell derived); HMGB, high mobility group box; hnMSC, human neonatal mesenchymal stem cells; hpHep, human primary hepatocytes; KC, Kupffer cells; MDA, malondialdehyde; miR, microRNA; MMP, mitochondrial membrane potential; MRP, multidrug resistance-associated protein; NA, not applicable; NAPQI, *N*-acetyl-*p*-benzoquinone imine; NPC, primary human non-parenchymal cells; OATP, organic-anion-transporting polypeptides; OCR, oxygen consumption rate; ROS, reactive oxygen species; rpHep, rat primary hepatocytes; SCSIT, cell spreading inhibition test; UGT, UDP-glucuronosyltransferase.

* in these reports, no IC_50_ was calculated and the values presented correspond to the concentration levels (mM) in which toxicity was observed.
