# Supplementary material for: A Critical Perspective on 3D Liver Models for Drug Metabolism and Toxicology Studies
Source: Front Cell Dev Biol. 2021 Feb 22;9:626805. doi: 10.3389/fcell.2021.626805 (PMC7957963; doi:10.3389/fcell.2021.626805)
Supplement: Supplementary file 2 [file Table_2.docx]

Table S2. Diclofenac cytotoxicity evaluation, metabolism assessment and mechanistic endpoints in different cell types and cell culture systems.

| **Cell Type** | **Cell Culture System** | **Exposure Time** | **IC_50_ / EC_50_ / LC_50_ / TC_50_ (µM)** | **Cytotoxicity Endpoints** | **Biotransformation \| Mechanistic Endpoints** | **References** |
| --- | --- | --- | --- | --- | --- | --- |
| FaO | 2D | 24h | 700 | MTT | Response to metabolism inhibitors \| Intracellular GSH, Ca^2+^ homoeostasis alteration, lipid peroxidation | (Ponsoda et al., 1995) |
| Huh7 | 2D | 24h | 686 | Alamar Blue | NA | (Lin et al., 2012) |
| HCCT-T | 2D | 24h | 104 | Alamar Blue | NA |  |
| HepG2 | 2D | 24h | 750 | MTT | Response to metabolism inhibitors \| Intracellular GSH, Ca^2+^ homoeostasis alteration, lipid peroxidation | (Ponsoda et al., 1995) |
|  |  |  | 399 | WST-1 | NA | (Wang et al., 2002) |
|  |  |  | ~1700 | ATP quantification | CYP activity, glucuronidation and sulfation activity, hepatobiliary transport \| NA | (Ramaiahgari et al., 2014) |
|  |  | 5 days (compound addition at D0 and 2) | 171.72 | Live cell protease activity | CYP activity, glucuronidation and sulfation activity \| NA | (Atienzar et al., 2014) |
|  | 3D Spheroids in dynamic culture | 24h | *500 | LDH, Glucose secretion, γ GT leakage, spheroid SCSIT | NA | (Xu et al., 2003) |
|  | 3D Spheroids with Matrigel | 24h  7 days | ~2000  ~400 | ATP quantification | CYP activity, glucuronidation and sulfation activity, hepatobiliary transport \| NA | (Ramaiahgari et al., 2014) |
| HepG2/C3A | 2D | 24h | >400 | ATP quantification | CYP2E1 expression \| NA | (Gaskell et al., 2016) |
|  | 3D Spheroids on Agarose Overlay | 24h | 295 | ATP quantification | CYP2E1 expression \| NA |  |
|  | 3D Spheroids | 4 days | 177.64 |  | NA \| BSEP inhibition, mitochondrial toxicity and bioactivation | (Williams et al., 2020) |
| Hep3B | 2D | 72h | 120 | MTT | NA | (Yu et al., 2018) |
|  | 3D Miniaturized cell-culture array (DataChip + Metachip) | 24h | 780 | Calcein-AM (live) / ethidium homodimer (dead) fluorescence |  |  |
|  | 3D Miniaturized cell-culture array (DataChip) + CYP450 enzymes (MetaChip) |  | 520 |  |  |  |
|  | 3D Miniaturized cell-culture array (DataChip) + CYP450 + phase II enzymes (MetaChip) |  | 790 |  |  |  |
|  | 3D Miniaturized cell-culture array (DataChip) + human liver microsomes (MetaChip) |  | 860 |  |  |  |
| HLCs  (hESC) | 2D | 24h | 1860 | MTT Assay | CYP activity \| NA | (Tasnim et al., 2015) |
|  |  | 1, 4 and 7 days | >200 |  |  | (Szkolnicka et al., 2014) |
| HLC  (hnMSC) | 2D | 24h | 1510 | MTS Assay | CYP and UGT activity, bupropion and diclofenac conversion \| NA | (Cipriano et al., 2017b) |
|  | 3D Spheroids | 24h | 980 |  |  |  |
| rpHep | 2D | 24h | 393 | MTT | Response to metabolism inhibitors \| Intracellular GSH, Ca^2+^ homoeostasis alteration, lipid peroxidation, gluconeogenesis | (Ponsoda et al., 1995) |
|  |  |  | 138 | ATP quantification | NA | (Lauer et al., 2009) |
|  |  |  | 263 | WST-1 | NA | (Wang et al., 2002) |
|  | 3D Collagen sandwich | 24h | 400 | Calcein AM (live) / PI (dead) fluorescent stain | CYP activity \| NA | (Zhang et al., 2011) |
|  | 3D Scaffold perfused bioreactor (RoboTox) |  | 180 |  |  |  |
|  | 3D Spheroids in dynamic culture |  | *500 | LDH, Glucose secretion, γ GT leakage, spheroid SCSIT | NA | (Xu et al., 2003) |
| hpHep | 2D | 24h | 1413 | Alamar Blue | NA | (Lin et al., 2012) |
|  | 3D Hollow-fiber Bioreactor | 7 days | *300 | Lactate secretion was decreased | NA | (Knöspel et al., 2016) |
|  |  |  | *1000 | Lactate secretion, cell density and cell organization were decreased; ammonia release was increased |  |  |
| Cryo hpHep | 2D | 24h | 222 | ATP quantification | NA | (Lauer et al., 2009) |
|  |  |  | 529.5 |  | NA \| Inflammatory response | (Li et al., 2020) |
|  |  |  | 3586 | MTT Assay | CYP activity \| NA | (Tasnim et al., 2015) |
|  |  |  | 250-500 | Live cell protease/caspase-3/7 | NA \| Mitochondrial dysfunction (OCR) | (Goda et al., 2016) |
|  |  | 48h | >4500 | ATP quantification | NA \| miR-122, HMGB1 and α-GST | (Proctor et al., 2017) |
|  |  | 5 days (compound addition at D0 and 2) | 100.01 | Live cell protease activity | CYP activity, glucuronidation and sulfation activity \| NA | (Atienzar et al., 2014) |
|  |  | 1, 4 and 7 days | >200 | MTT Assay | CYP activity \| NA | (Szkolnicka et al., 2014) |
|  | 3D Spheroids | 48h  7 days  28 days | 191  57  46 | ATP quantification | CYP activity \| Bile acid and neutral lipids accumulation, viral infection | (Bell et al., 2016) |
|  |  | 24h after repeated dosing at D8, 12 and 15 | 64.2 |  | NA \| Inflammatory response | (Li et al., 2020) |
| Co-culture of dog hepatocytes and NPC | 2D | 5 days (compound addition at D0 and 2) | 205.7 | Live cell protease activity | CYP activity, glucuronidation and sulfation activity \| NA | (Atienzar et al., 2014) |
| Co-culture of cryo hpHep and NPC | 3D Spheroid human liver microtissues (3D hLiMT) | 14 days | 178.6 | ATP quantification | NA | (Messner et al., 2013) |
|  |  | 5-6 days  14 days | 100.0  61.4 |  | NA \| miR-122, HMGB1 and α-GST | (Proctor et al., 2017) |
| Co-culture of hpHep and KC | LiverChip bioreactors | 48h | 227 | WST-1 | CYP and UGT activity, metabolite identification, diclofenac protein binding and clearance \| Bile acid identification and quantification, inflammatory response | (Sarkar et al., 2017) |
|  | 3D Spheroids | 5 days | NA | ATP quantification | NA \| Inflammatory response | (Li et al., 2020) |

BSEP, bile salt export pump; Ca^2+^, calcium; cryo, cryopreserved; CYP, cytochrome P450; GSH, glutathione; GST, glutathione S-transferase; HepG2, HepG2/C3A, FaO, Huh7, HCCT-T, hepatic cell lines; hESC, human embryonic stem cells; HLC, hepatocyte-like cells (stem cell derived); HMGB, high mobility group box; hnMSC, human neonatal mesenchymal stem cells; hpHep, human primary hepatocytes; KC, Kupffer cells; miR, microRNA; NA, not applicable; NPC, primary human non-parenchymal cells; OCR, oxygen consumption rate; rpHep, rat primary hepatocytes; SCSIT, cell spreading inhibition test; UGT, UDP-glucuronosyltransferase.

* in these reports, no IC_50_ was calculated and the values presented correspond to the concentration levels (µM) in which toxicity was observed.
