# Supplementary material for: A Critical Perspective on 3D Liver Models for Drug Metabolism and Toxicology Studies
Source: Front Cell Dev Biol. 2021 Feb 22;9:626805. doi: 10.3389/fcell.2021.626805 (PMC7957963; doi:10.3389/fcell.2021.626805)
Supplement: Supplementary file 3 [file Table_3.docx]

Table S3. Troglitazone cytotoxicity evaluation in different cell types and cell culture systems.

| **Cell Type** | **Cell Culture System** | **Exposure Time** | **IC_50_ / EC_50_ / LC_50_ / TC_50_ (µM)** | **Cytotoxicity Endpoints** | **Biotransformation \| Mechanistic Endpoints** | **References** |
| --- | --- | --- | --- | --- | --- | --- |
| HepG2 | 2D | 5 days (compound addition at D0 and 2) | 95.85 | Live cell protease activity | CYP activity, glucuronidation and sulfation activity \| NA | (Atienzar et al., 2014) |
|  |  | 24h | ~200 | ATP quantification | CYP activity, glucuronidation and sulfation activity, hepatobiliary transport \| NA | (Ramaiahgari et al., 2014) |
|  | 3D  Spheroids with Matrigel | 24h after 21 days in culture  7 days after 21 days in culture | ~200  ~80 |  |  |  |
| HepG2/C3A | 3D Spheroids | 4 days | 42.71 | ATP quantification | NA \| BSEP inhibition, mitochondrial toxicity and bioactivation | (Williams et al., 2020) |
| HepaRG | 2D | 24h after 5 days in culture  24h after 22 days in culture | 41  301.3 | ATP quantification | CYP2E1 and MRP-2 activity \| NA | (Gunness et al., 2013) |
|  |  | 48h  7 days  14 days | >100  36.5  34.6 |  | *CYP, GSTT1, UGT1A1, ABCB11, ABCC1* and *SLCO1B1* gene expression \| NA | (Bell et al., 2017) |
|  | 3D Spheroids | 24h after 5 days in culture  24h after 22 days in culture | 398  >500 | ATP quantification | CYP2E1 and MRP-2 activity \| NA | (Gunness et al., 2013) |
|  |  | 8 days  14 days | ~15  ~10 |  | NA \| BSEP inhibition, bile acid accumulation, F-actin cytoskeleton disruption | (Hendriks et al., 2016) |
| HLCs  (hESC) | 2D | 24h | 33.7 | MTT Assay | CYP activity \| NA | (Tasnim et al., 2015) |
|  |  | 24h  4 days  7 days | 198.53  101.06  85.74 | ATP quantification | CYP activity \| NA | (Szkolnicka et al., 2014) |
| HLCs  (hiPSC) | 2D | 48h  7 days  14 days | 46  33.9  18.7 | ATP quantification | *CYP, GSTT1, UGT1A1, ABCB11, ABCC1* and *SLCO1B1* gene expression \| NA | (Bell et al., 2017) |
| rpHep | 2D | 24h | 140 | ATP quantification | NA | (Lauer et al., 2009) |
|  |  | 48h | *6 | MTT assay  LDH leakage | CYP3A4 activity \| GSH content, MDA and MMP potential measurement, lipid accumulation | (Shen et al., 2012) |
|  | 3D Gel entrapment | 48h  21 days | No toxic response  *30 |  |  |  |
| hpHep | 3D Gel entrapment | 4 days | *6 | MTT assay  LDH leakage | CYP3A4 activity \| GSH content, MDA and MMP potential measurement, lipid accumulation | (Shen et al., 2012) |
| Cryo hpHep | 2D | 24h | 62.5-125 | Live cell protease/  caspase-3/7 | NA \| Mitochondrial dysfunction (OCR) | (Goda et al., 2016) |
|  |  |  | 29.7 | MTT Assay | CYP activity \| NA | (Tasnim et al., 2015) |
|  |  |  | 30.9 | ATP quantification | NA \| inflammatory response | (Li et al., 2020) |
|  |  |  | 88 |  | NA | (Lauer et al., 2009) |
|  |  | 24h  4 days  7 days | >200  >200  37.93 |  | CYP activity \| NA | (Szkolnicka et al., 2014) |
|  |  | 48h | >4500 |  | NA \| miR-122, HMGB1 and α-GST | (Proctor et al., 2017) |
|  |  | 5 days (compound addition at D0 and 2) | 55.59 | Live cell protease activity | CYP activity, glucuronidation and sulfation activity \| NA | (Atienzar et al., 2014) |
|  | 3D Spheroids | 24h after repeated dosing at D8, 12 and 15 | 1.0 | ATP quantification | NA \| inflammatory response | (Li et al., 2020) |
|  |  | 8 days  14 days | ~ 8.0  ~7.8 |  | NA \| BSEP inhibition, bile acid accumulation, F-actin cytoskeleton disruption | (Hendriks et al., 2016) |
|  |  | 48h  7 days  14 days | 37.4  4.2  1.5 |  | *CYP, GSTT1, UGT1A1, ABCB11, ABCC1* and *OATP-C* gene expression \| NA | (Bell et al., 2017) |
| Co-culture of rpHep and NPC | 2D | 48h | *from 10-100 µM | ATP quantification  LDH leakage | CYP activity \| NA | (Kostadinova et al., 2013) |
|  | 3D Nylon scaffold | 1 to 8 days | No marked toxicity |  |  |  |
| Co-culture of dog hepatocytes and NPC | 2D | 5 days (compound addition at D0 and 2) | 57.51 | Live cell protease activity | CYP activity, glucuronidation and sulfation activity \| NA | (Atienzar et al., 2014) |
| Co-culture of hpHep and NPC | 2D | 48h | No marked toxicity | ATP quantification  LDH leakage | CYP activity \| NA | (Kostadinova et al., 2013) |
|  | 3D Nylon scaffold | 1 to 8 days | *from 10-100 µM |  |  |  |
| Co-culture of cryo hpHep and NPC | 3D Spheroid human liver microtissues (3D hLiMT) | 5-6 days  14 days | 25.6  14.6 | ATP quantification | NA \| miR-122, HMGB1 and α-GST | (Proctor et al., 2017) |
| Co-culture of cryo hpHep and KC | 3D Spheroids | 5 days | *1.0 | ATP quantification | NA \| inflammatory response | (Li et al., 2020) |

ABC, ATP Binding Cassette; BSEP, bile salt export pump; cryo, cryopreserved; CYP, cytochrome P450; ECAR, extracellular acidification rates; GSH, glutathione; GST, glutathione S-transferase; HepG2, HepaRG, HepG2/C3A, hepatic cell lines; hESC, human embryonic stem cells; HMGB, high mobility group box; hpHep, human primary hepatocytes; KC, Kupffer cells; MDA, malondialdehyde; MMP, mitochondrial membrane potential; mpHep, mouse primary hepatocytes; MRP, multidrug resistance-associated protein; NA, not applicable; NPC, non-parenchymal cells; OATP, organic-anion-transporting polypeptides; OCR, oxygen consumption rate; rpHep, rat primary hepatocytes; UGT, UDP-glucuronosyltransferase.

* in these reports, no IC_50_ were calculated and the values presented correspond to the concentration levels (µM) in which toxicity was observed.
